# Intratumoral microbiota in cancer: molecular mechanism and therapeutic strategies

**DOI:** 10.1186/s43556-026-00473-w

**Published:** 2026-05-22

**Authors:** Yao Yu, Ziyu Guo, Zehao Luo, Yating Dian, Xiaoxin Yang, Xiang Chen, Furong Zeng, Guangtong Deng

**Affiliations:** 1 Department of Dermatology, Xiangya Hospital, Central South University, Changsha, China; 2 Hunan Engineering Research Center of Skin Health and Disease, Central South University, Changsha, China; 3 Hunan Key Laboratory of Skin Cancer and Psoriasis, Central South University, Changsha, China; 4 National Engineering Research Center of Personalized Diagnostic and Therapeutic Technology, Changsha, China; 5 National Clinical Research Center of Geriatric Disorders, Xiangya Hospital, Central South University, Changsha, China; 6 Hunan Key Laboratory of Aging Biology, Changsha, China; 7 Department of Oncology, Xiangya Hospital, Central South University, Changsha, Hunan Province 410008 China

**Keywords:** Intratumoral microbiota, Tumor microenvironment, Host microbial interactions, Therapeutics, Microbial regulation, Analysis methods

## Abstract

Recent spatially resolved, multi-omic, and functional studies have advanced understanding of the intratumoral microbiota (ITM), shifting attention from microbial detection in tumors to how microbial localization and host-cell interactions influence cancer phenotypes and therapeutic outcomes. In this review, we first trace the historical development of the field and synthesize current evidence on routes of microbial colonization, pan-cancer heterogeneity, and the spatial organization of ITM. We then discuss how ITM directly affects genomic stability, signaling pathways, metabolism, and cellular plasticity of cancer cells, while indirectly shaping tumor evolution through its effects on immune and stromal components within the tumor microenvironment (TME). We further assess the role of ITM in anticancer therapy, explicitly distinguishing mechanisms supported by direct evidence in tumors from those inferred primarily from gut microbiota-driven systemic effects. In addition, we summarize emerging strategies to target or exploit ITM, including antibiotics, phage strategy, engineered strategy and related microbiota-modulating interventions. Finally, we highlight the major challenges that continue to constrain the field, particularly low microbial biomass, contamination, limited spatial resolution, and insufficient in vivo functional validation. Together, these considerations position ITM as a context-dependent component of tumor ecosystems with potential relevance to tumor progression, therapeutic stratification, and biomarker development, while defining priorities for more rigorous and clinically actionable research.

## Introduction

Cancer is now widely recognized not merely as a disease of mutated cells, but as a complex, spatially organized ecosystem shaped by dynamic interactions within the tumor microenvironment (TME) [1, 2]. Historically, solid tumors were generally assumed to be sterile. However, the identification of persistent, tumor-type-specific microorganisms residing within cancer cells and the surrounding stroma has fundamentally disrupted this paradigm, establishing the intratumoral microbiota (ITM) as a crucial new dimension of cancer biology [3–6]. These observations have raised growing interest in the origin, composition, and potential functional significance of ITM.

At the same time, the field remains difficult to synthesize into a coherent framework. One reason is that discussions of ITM are frequently interwoven with findings derived from the gut microbiota and its systemic immune effects, even though these may reflect different biological settings and mechanistic layers [7–11]. In addition, differences in tumor type, sampling strategies, and analytical platforms have generated heterogeneous datasets that further complicate cross-study comparison and biological interpretation [11, 12]. These limitations have delayed the translation of ITM research into actionable clinical strategies. Therefore, a more integrated and critical synthesis is urgently needed.

In this review, we aim to organize this rapidly expanding literature through a framework, which explicitly distinguishes the phenomena supported by direct intratumoral evidence from those inferred through gut-mediated systemic host responses. We first outline the historical evolution of tumor microbiology and examine the potential anatomical routes and factors driving microbial colonization within tumors. We then delve into the mechanistic functions of the ITM, exploring its direct effects on malignant cells and its indirect role in remodeling the TME. Next, we map the diversity of the ITM across various cancer types and critically evaluate its impact on therapeutic efficacy, with particular attention to the distinction between intratumoral mechanisms and systemic microbiome effects. Finally, we highlight innovative strategies for therapeutically targeting or exploiting the ITM, and discuss the critical methodological and translational challenges that must be addressed before these approaches can support personalized cancer management.

## Major landmarks of intratumoral microbiota

**Fig. 1:**
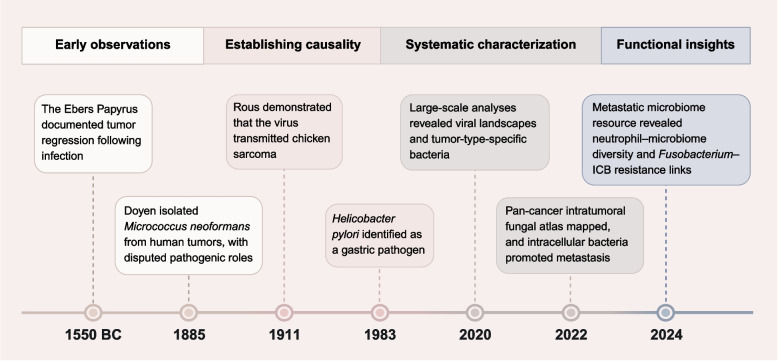
Major landmarks of intratumoral microbiota. The timeline summarizes key milestones in the field, spanning early observations, the establishment of microbial causality in cancer, systematic characterization of intratumoral microbiota, and recent functional insights. Abbreviations: ICB, immune checkpoint blockade

The association between microbes and cancer has been recorded for millennia, laying the earliest foundation for the field of ITM research (Fig. 1). Ancient records such as the Ebers Papyrus (1550 BC) documented tumor regression following infection [13]. In the thirteenth century, Peregrine Laziosi’s recovery from an infected tibial tumor was among the first anecdotal accounts suggesting a link between infection and cancer control [14]. The first direct isolation of microbes from tumors was reported in 1885, when Doyen isolated *Micrococcus neoformans* from human tumors, offering early evidence of microbial presence within tumors, though its pathogenic role was disputed [15].

The causal relationship between microbes and cancer began to crystallize in the early twentieth century. The modern era of tumor microbiology began with Peyton Rous’s seminal 1911 demonstration that a filterable agent (later identified as Rous sarcoma virus) could transmit cancer in chickens, providing the first experimental evidence of viral oncogenesis [16]. This was followed by the discovery of other oncogenic viruses, including Epstein-Barr virus (EBV), Kaposi’s sarcoma-associated herpesvirus (KSHV), human papillomavirus (HPV), hepatitis B and C viruses (HBV, HCV), and Merkel cell polyomavirus (MCPyV) [17–19]. A breakthrough came in 1983, when Warren and Marshall identified *Helicobacter pylori* (*H. pylori*) as a cause of gastritis and gastric ulcers, later recognized as a Group I carcinogen for gastric cancer (GC) [20–23]. This discovery expanded tumor microbiology research beyond oncogenic viruses to bacterial tumorigenesis.

The advent of high-throughput sequencing technologies, such as 16S ribosomal RNA (rRNA) and shotgun metagenomics, propelled the field into a new era of systematic characterization of ITM communities. In 2020, Lichter et al. conducted a landmark pan-cancer study across 38 tumor types, which systematically defined the intratumoral viral landscape, revealed canonical oncovirus prevalence, and critical virus-driven oncogenic mechanisms [5]. That same year, Straussman’s group revealed tumor-type-specific bacterial communities within cancer cells and immune cells, with predicted bacterial metabolic functions associated with certain tumor subtypes and clinical features [4]. In 2022, the same team mapped fungal communities across 35 cancer types, reporting co-occurrence with tumor-associated bacteria and links to immune responses [6], while Iliev et al. published a pan-cancer fungal atlas linking fungal taxa, particularly Candida species, to gastrointestinal and lung tumors, inflammatory immune pathways, metastasis, and reduced survival [24].

Another key milestone focused on elucidating the functional roles of ITM in tumor progression, particularly in metastasis, the most lethal hallmark of cancer. In 2022, Cai’s group showed in 2022 that tumor-resident intracellular bacteria carried by circulating tumor cells promoted metastatic colonization in breast cancer (BC) by enhancing tumor-cell survival under fluid shear stress. Notably, depletion of intratumoral bacteria reduced lung metastasis without affecting primary tumor growth [25]. Battaglia et al. established a large-scale resource of the metastatic tumor microbiome in 2024 through integrated multi-omics analyses of metastatic tumor samples. The study revealed site-specific microbial patterns and associations between microbial diversity and tumor-infiltrating neutrophils, including a link between *Fusobacterium* and resistance to immune checkpoint blockade (ICB) in lung cancer (LC) [26].

Overall, ITM research has evolved from early clinical observations and pathogen discovery to pan-cancer multi-omic mapping and functional validation. This transition has established ITM as a biologically meaningful component of the TME, and has supported further research into its clinical relevance, mechanistic contributions, and potential as a biomarker and therapeutic target in cancer.

## Routes of intratumoral microbiota colonization

**Fig. 2:**
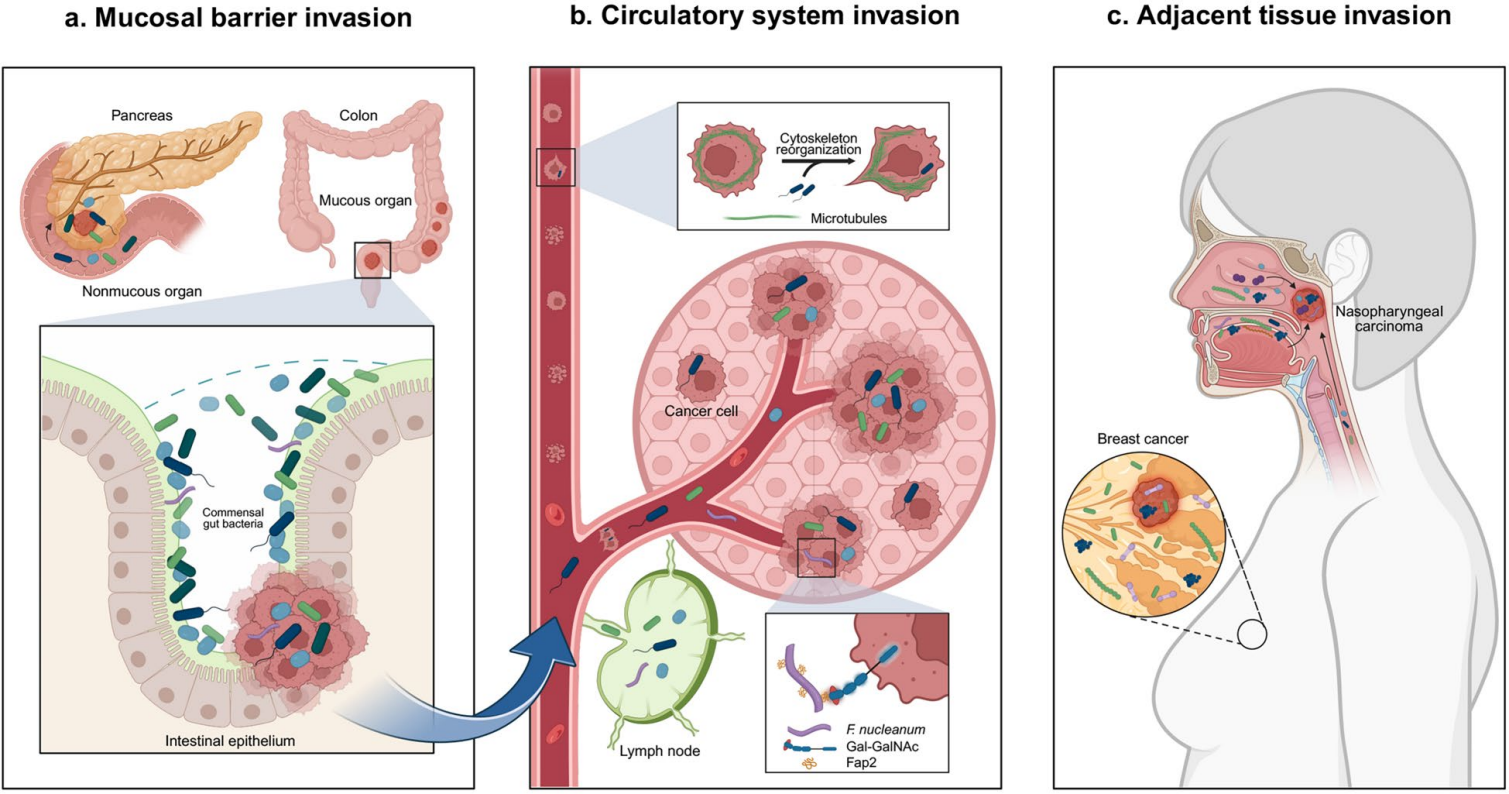
Origin of intratumoral microbiota. **a** Mucosal barrier invasion. Commensal gut bacteria may penetrate damaged mucosal barriers and translocate into nonmucosal organs. **b** Circulatory system invasion. Microorganisms can enter the bloodstream, survive within or adhere to host cells, and reach distant tumors through hematogenous spread. *Staphylococcus* and *Lactobacillus* remodel the actin cytoskeleton via RhoA-ROCK signaling, increasing resistance to shear stress. *Fusobacterium nucleatum* mediates adhesion to tumor cells via its Fap2 protein, which binds to Gal-GalNAc. **c** Adjacent tissue invasion. Microorganisms may directly infiltrate tumors from the neighboring, contiguous normal tissue, with which the tumor often shares a similar microbial community. Abbreviations: *F. nucleatum*, *Fusobacterium nucleatum*; GalNAc, N-acetylgalactosamine; Fap2, fibroblast activation protein-2

Understanding how ITM gains access to tumor tissues is essential for interpreting its diverse biological effects and compositional variation across tumor types. Current evidence suggests that ITM colonization occurs through three primary routes: (1) mucosal barrier invasion; (2) circulatory system invasion; and (3) adjacent tissue invasion (Fig. 2).

Mucosal surfaces, including the gastrointestinal, respiratory, and genitourinary tracts, are densely colonized by commensals. Barrier disruption, an early event in tumor initiation, facilitates microbial invasion. For example, *Ascomycota*, *Bacteroidetes*, and *Cyanobacteria* detected in pancreatic ductal adenocarcinoma (PDAC) are thought to originate from the duodenum and migrate retrogradely via the pancreatic duct [3, 27]. Similarly, oral gavage of *Enterococcus faecalis* leads to its enrichment in pancreatic tissue [28]. In bladder cancer (BCa), recurrent *Escherichia coli* (*E. coli*) urinary tract infections promote colonization and contribute to oncogenesis [29].

Circulatory system invasion is a key route for ITM colonization. *Lactobacillus reuteri* (*L. reuteri*) can reach melanoma via vascular and lymphatic circulation [7]. Gut-derived *E. coli* disrupts the gut-vascular barrier (GVB) and translocates to the liver, forming pre-metastatic niches [30]. *Enterococcus hirae* (*E. hirae*) migrates to secondary lymphoid organs during immunotherapy [31, 32]. *Fusobacterium nucleatum* (*F. nucleatum*) targets colorectal tumors via its Fap2 protein binding to circulating Gal-GalNAc receptors [33]. Moreover, microbial similarity between primary and metastatic lesions suggests co-migration with cancer cells [34]. Consistent with this view, intracellular bacteria have been detected within tumors and immune cells, and circulating tumor cells can carry bacteria that promote metastasis through cytoskeletal remodeling [4, 25].

Normal adjacent tissues (NATs) also serve as microbial reservoirs. Microbial communities in BC and glioblastoma (GBM) closely resemble those in paired normal tissues [4]. In nasopharyngeal carcinoma (NPC), microbial profiles overlap significantly with nasopharyngeal (69.0%), oral (24.1%), and intestinal (6.9%) microbiota, suggesting local migration [35]. *F. nucleatum* enrichment in tongue cancer implies oral origin [36], and spatial transcriptomics in LC indicates microbial gradients decreasing from small airways to tumor core, suggesting anatomically guided dissemination [37]. However, whether NAT microbiota originate from tumors or vice versa requires further investigation.

## The dual role of intratumoral microbiota in tumor

**Fig. 3:**
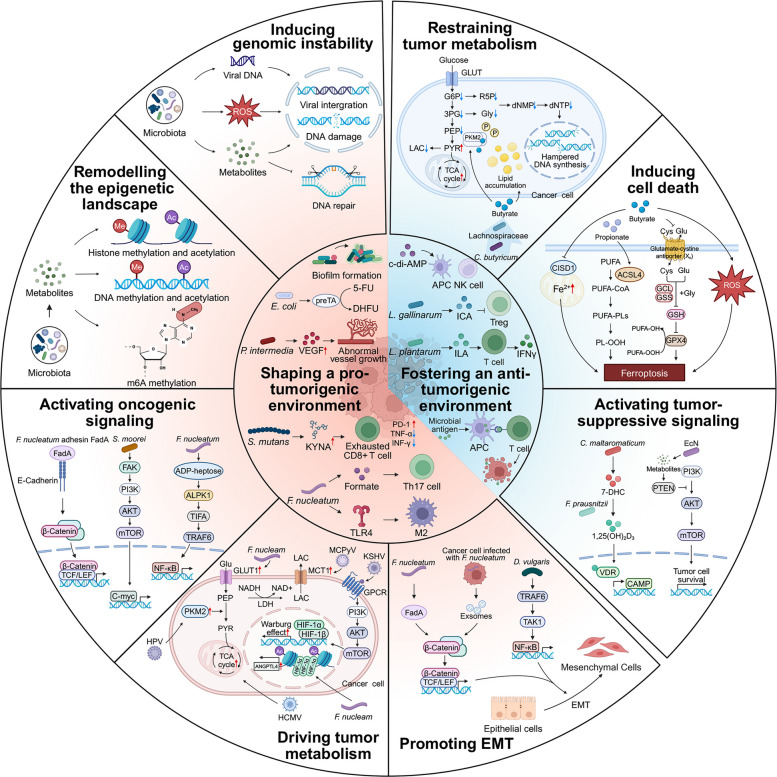
The dual role of intratumoral microbiota in tumor. Protumor activities include inducing genomic instability (viral integration and bacterial genotoxins), remodelling the epigenetic landscape (microbe-driven DNA, histone, and RNA modifications), activating oncogenic signaling (WNT/β-catenin pathway, PI3K/AKT/mTOR pathway, NF-κB pathway, etc.), driving tumor metabolism, promoting EMT, and shaping a pro-tumorigenic microenvironment (biofilms, microbial drug metabolism, angiogenesis, and immune evasion). Antitumor activities include restraining tumor metabolism, inducing cancer cell death, activating tumor-suppressive signaling (VDR activation and AKT/mTOR inhibition), and fostering an anti-tumor immune environment (immune activation). Abbreviations: 5-FU, 5-fluorouracil; DHFU, dihydrofluorouracil; *F. nucleatum, Fusobacterium nucleatum*; *Helicobacter pylori*; *S. moorei, Solobacterium moorei*; FadA, Fusobacterium adhesin A; FAK, focal adhesion kinase; C-myc, cellular myelocytomatosis oncogene; ADP-heptose, adenosine diphosphate heptose; ALPK1, alpha kinase 1; TIFA, TRAF-interacting protein with forkhead-associated domain; TRAF6, TNF receptor-associated factor 6; VEGF, vascular endothelial growth factor; KYNA, kynurenic acid; TLR4, Toll-like receptor 4; TAK1, transforming growth factor-β-activated kinase 1; LAC, lactate; GLUT1, glucose transporter 1; NAD, nicotinamide adenine dinucleotide; LDH, lactate dehydrogenase; HIF-1α, hypoxia-inducible factor 1-alpha; MCT1, monocarboxylate transporter 1; PI3K, phosphoinositide 3-kinase; AKT, protein kinase B; mTOR, mechanistic target of rapamycin; EMT, epithelial–mesenchymal transition; APC, antigen-presenting cell; NK cell, natural killer cell; Treg, regulatory T cell; Th17, T helper 17 cell; VDR, vitamin D receptor; PUFA, polyunsaturated fatty acid; ROS, reactive oxygen species; TCA, tricarboxylic acid; m6A, N6-methyladenosine; Glu, glutamate; PKM2, pyruvate kinase M2; *D. vulgaris, Desulfovibrio vulgaris*; *C. maltaromaticum*, *Carnobacterium maltaromaticum*; EcN, *Escherichia coli Nissle* 1917; *C. butyricum, Clostridium butyricum*; *E. coli, Escherichia coli*; *P. intermedia, Prevotella intermedia*; *L. gallinarum, Lactobacillus gallinarum*; *L. plantarum, Lactobacillus plantarum*; MCPyV, Merkel cell polyomavirus; KSHV, Kaposi’s sarcoma-associated herpesvirus; NADH, nicotinamide adenine dinucleotide (reduced form); NAD +, nicotinamide adenine dinucleotide (oxidized form); Ac, acetylation; ANGPTL4, angiopoietin-like 4; GPCR, G protein-coupled receptor; HPV, human papillomavirus; HCMV, human cytomegalovirus; HIF-1β, hypoxia-inducible factor 1 beta; TCF, T cell factor; LEF, lymphoid enhancer-binding factor; 7-DHC, 7-dehydrocholesterol; 1,25(OH)₂D₃, 1,25-dihydroxyvitamin D₃; CAMP, cathelicidin antimicrobial peptide; PTEN, phosphatase and tensin homolog; CISD1, CDGSH iron sulfur domain 1; ACSL4, acyl-CoA synthetase long-chain family member 4; PUFA-CoA, polyunsaturated fatty acyl-coenzyme A; PUFA-PLs, polyunsaturated phospholipids; PL-OOH, phospholipid hydroperoxide; PUFA-OH, hydroxylated polyunsaturated fatty acid; PUFA-OOH, polyunsaturated fatty acid hydroperoxide; GSH, glutathione; GPX4, glutathione peroxidase 4; GSS, glutathione synthetase; GCL, glutamate-cysteine ligase; Gly, glycine; Cys, cysteine; G6P, glucose-6-phosphate; 3PG, 3-phosphoglycerate; PEP, phosphoenolpyruvate; PYR, pyruvate; R5P, ribose-5-phosphate; dNMP, deoxynucleoside monophosphate; dNTP, deoxynucleoside triphosphate; PD-1, programmed cell death protein 1; TNF-α, tumor necrosis factor alpha; IFN-γ, interferon gamma; M2, M2 macrophage; c-di-AMP, cyclic di-adenosine monophosphate; ICA, indole-3-carboxylic acid; ILA, indole-3-lactic acid

Once established within tumors, ITM can exert bidirectional effects on tumors, with the potential to either promote or restrain malignant progression. Its localized mechanisms encompass both direct effects on tumor cells, including impaired genomic stability, dysregulated signaling pathways, reprogrammed metabolism, and enhanced migratory capacity, and indirect effects mediated through remodeling of the TME. Elucidating these intrinsic mechanisms through which ITM governs tumorigenesis and progression is therefore critical for advancing precision medicine (Fig. 3).

### Pro-tumorigenic functions of intratumoral microbiota

#### Inducing genomic instability

ITM promotes tumorigenesis by inducing DNA damage and impairing repair mechanisms. Oncogenic viruses such as HPV and HBV integrate into host genomes, producing oncoproteins and dysregulating host signaling [6, 38, 39], while bacterial genotoxins drive genomic instability. Polyketide synthase-positive *Escherichia coli* (pks + *E. coli*)-derived colibactin and metabolites from oral microorganisms such as *Streptococcus salivarius* and *Neisseria* generate DNA adducts that lead to genomic damage and mutagenesis [40–42], effects enhanced by co-colonization with *enterotoxigenic Bacteroides fragilis* (ETBF) and dependent on epithelial adhesion mediated by FimH and FmlH [43]. Additional toxins, including cytotoxic necrotizing factor 1 (CNF1) and UDP-sugar hydrolase from pathogenic *E. coli*, induce reactive oxygen species (ROS) and DNA lesions [44, 45]. ETBF also induces spermine oxidase-dependent ROS production and γ-H2A.x activation [46]. Chronic *F. nucleatum* infection similarly generates ROS to damage DNA [47]. Mycotoxins, such as aflatoxins produced by *Aspergillus flavus*, form mutagenic DNA adducts [48, 49]. Beyond direct DNA damage, microorganisms also impair DNA repair mechanisms. Aflatoxin adducts inhibit nucleotide excision repair, viral proteins such as human T lymphotropic virus type-1 Tax and MCPyV disrupt repair signaling, and pathogens, including EspF-expressing enteropathogenic *E. coli* and *P. gingivalis*, compromise mismatch repair, collectively exacerbating genomic instability [50–54]. This mechanism explains why colonization of specific microbes is closely linked to early tumor initiation and mutation burden, providing novel microbial biomarkers for early tumor screening and prevention.

#### Remodelling the epigenetic landscape

In addition to causing DNA damage, ITM regulates host gene expression through epigenetic modifications such as DNA methylation, histone modifications, and non-coding RNAs, thereby further contributing to tumor initiation and progression.[55] Among these, aberrant DNA methylation is frequent: *H. pylori*, *Kytococcus sedentarius*, and *Actinomyces oris* induce DNA methylation in gastric tissues, while *F. nucleatum* and *Hungatella hathewayi* upregulate DNA methyltransferases in colorectal cancer (CRC), silencing tumor suppressors such as CDX2 and MLH1 [56–58]. *F. nucleatum* also induces DNA hypermethylation via an MSH2/MSH6-dependent DNA repair mechanism [47]. CpG island methylator phenotype-positive CRCs are enriched in *Bacteroides fragilis* (*B. fragilis*), *F. nucleatum*, and *Klebsiella pneumoniae* (*K. pneumoniae*), closely linked to CpG island methylator phenotype and MLH1 methylation [59]. Dysregulated histone modifications also contribute, exemplified by *F. nucleatum*-induced lncRNA ENO1-IT1, which recruits KAT7 to reprogram histones [60]. Microbial metabolites, including short-chain fatty acids (SCFAs), folate, taurocholic acid, vitamins, and S-adenosylmethionine, regulate DNA and histone acetylation and methylation [61]. RNA modifications further shape oncogenesis, as *F. nucleatum* enhances METTL3 expression, leading to N6-methyladenosine (m6A) methylation and stabilization of c-myc mRNA [62], and HBV also modifies m6A to support tumor progression [63]. Such epigenetic remodeling may help stabilize malignant phenotypes while preserving the plasticity needed for tumor progression.

#### Activating oncogenic signaling

ITM activates diverse oncogenic signaling pathways, thereby modulating essential processes such as cell proliferation, metabolism, inflammation, and immune regulation. The WNT/β-catenin pathway, governing polarity growth and stemness maintenance, is aberrantly activated in various tumors. *H. pylori* CagA directly engages β-catenin to promote GC [64]. *Fusobacterium periodonticum* drives epithelial-mesenchymal transition (EMT) via PI3K-AKT/FASN signaling, enhancing WNT3A palmitoylation and β-catenin translocation in squamous cell carcinoma (ESCC) [65]. *F. nucleatum* adhesin FadA and *B. fragilis* toxin both disrupt E-cadherin to activate β-catenin in CRC [66]. Similarly, *Salmonella typhi* effector avirulence protein A and *Fusobacterium mortiferum* metabolite 5-aminovaleric acid promote β-catenin activation [67, 68]. Beyond gastrointestinal tumors, localized ITM-derived butyrate activates the β-catenin pathway and facilitates osteosarcoma metastasis [69]. The MAPK/ERK cascade contributes to tumor initiation via proliferation control. *P. gingivalis* promotes CRC cell proliferation through MAPK-ERK signaling and induces oral squamous cell carcinoma (OSCC) by activating ERK1/2-ETS1 and related axes [42, 70]. The PI3K/AKT/mTOR pathway is broadly implicated in cell survival, growth, and metabolic regulation. *S. typhimurium* and *Salmonella typhi* activate this pathway and drive gallbladder epithelial transformation [71]. *P. gingivalis* in gingival carcinoma triggers PI3K/AKT activation and suppresses mitochondrial apoptosis [42]. *Solobacterium moorei* promotes tumor progression through binding to integrin α2/β1 on CRC cells, thereby activating the PI3K-AKT-mTOR-C-myc axis [72]. In LC, enrichment of *Veillonella*, *Prevotella*, and *Streptococcus* in the lower airways correlates with PI3K pathway upregulation [73]. The JAK-STAT axis regulates cell cycle progression and apoptosis [74]. *P. gingivalis* promotes gingival squamous carcinoma via JAK1/STAT3 activation [42]. In GC, *H. pylori* infection induces STAT3 Ser727 phosphorylation with mitochondrial localization, resulting in mitochondrial damage and autophagy [75]. ETBF induces Th17 responses and selectively activates STAT3 in Apc^min/+^ mice, contributing to colon tumorigenesis [76]. The NF-κB pathway serves as a pivotal link between inflammation and tumor development. *F. nucleatum* enhances RAS signaling, IL-8 expression, and chemoresistance via TLR4/MYD88/NF-κB, ALPK1/TIFA/TRAF6 axes, and TLR4-NF-κB-BIRC3 [77–79] while *Peptostreptococcus anaerobius* recruits immunosuppressive myeloid-derived suppressor cells (MDSCs) through integrin-NF-κB signaling [80]. *B. fragilis* toxin triggers MAPK and NF-κB, promoting neutrophil infiltration [81]. Beyond these canonical pathways, *Malassezia* glycans activate C3 convertase and promote tumor proliferation, motility, and invasion via C3a-C3aR signaling [82]. Across these studies, a common pattern is that ITM engages canonical oncogenic pathways and amplifies malignant programs already central to tumor cell fitness. Its convergence on pathways such as WNT/β-catenin, PI3K-AKT, JAK-STAT, and NF-κB suggests that diverse microbes may promote tumor progression through a limited set of high-value signaling nodes.

#### Driving tumor metabolism

ITM profoundly reprograms host metabolism, including carbohydrate, amino acid, nucleotide, and lipid metabolism. *F. nucleatum* enhances glycolysis by upregulating glucose transporter 1, lactate accumulation, and ANGPTL4 expression, while also rewiring lipid metabolism to support stemness and Notch signaling [83–85]. Viral oncoproteins further drive metabolic shifts: HPV E7 promotes PKM2 dimerization, MCPyV upregulates lactate transporter monocarboxylate transporter 1, and KSHV activates PI3K-AKT-mTOR-HIF-1α to enhance glycolysis [86–88]. Human cytomegalovirus simultaneously activates the glycolytic pathway, the tricarboxylic acid (TCA) cycle, and fatty acid biosynthesis [89, 90]. Beyond the localized metabolic reprogramming by ITM, global metabolic shifts driven by the gut microbiota also systematically support tumor progression. For instance, in cervical cancer (CC), non-*Lactobacillus* dominant gut microbial communities influence systemic amino acid and nucleotide metabolism [91], whereas in GC, metabolomic profiling shows alterations in carbohydrates, amino acids, and lipids associated with *H. pylori* depletion and *Lactobacillus* enrichment [92]. Nevertheless, distinguishing direct intratumoral effects from broader host–microbiome metabolic influences remains essential for precise mechanistic interpretation.

#### Promoting EMT

ITM facilitates tumor dissemination by promoting EMT, a process orchestrated by TGF-β/SMAD, PI3K/AKT, WNT/β-catenin, and MAPK pathways [93]. *F. nucleatum* promotes metastasis by secreting exosomes carrying chemokines and miRNAs, activating ERK signaling, and inducing EMT through upregulation of CCL2, CYP2J2, and YTHDF1, together with TLR4 activation [62, 94–97]. *Desulfovibrio vulgaris* flagellin engages the transmembrane receptor LRRC19 to activate TRAF6-TAK1 signaling, thereby driving EMT and enhancing proliferation, migration, and invasion in CRC models [98]. Tumor-resident *B. fragilis* activates Notch1 via SusD/RagB, inducing EMT and stemness [99]. *H. pylori* CagA induces EMT-like changes and stemness marker CD44 [100]. Microbial regulation of EMT-related transcription factors and markers further facilitates invasion. In BCa, *E. coli* strain SM4/1 and Oscillatoria alter the expression of EMT-related genes, including E-cadherin, vimentin, SNAI2, SNAI3, and TWIST1 [101]. *P. gingivalis* increases levels of Slug, Snail, Zeb1, and phosphorylated GSK-3β, fostering migration of oral epithelial cells [102]. Targeting microbe-associated EMT may offer a promising strategy to limit tumor metastasis and improve prognosis in patients with advanced cancer.

#### Shaping a pro-tumorigenic environment

In addition to directly reprogramming tumor cells, ITM can also influence cancer progression indirectly by reshaping the TME, particularly through immune suppression, inflammatory amplification, and niche maintenance.

A major component of this indirect effect is the suppression of adaptive antitumor immunity. *Nevskia ramosa*, *Staphylococcus aureus* (*S. aureus*), HBV, and HCV induce Treg-mediated suppression, facilitating prostate and liver cancer progression [103–105]. HPV suppresses cytotoxic T and natural killer (NK) cell activation by downregulating antigen presentation and upregulating PD-L1 via its E7 protein [106]. *F. nucleatum* and *H. pylori* engage TIGIT and CEACAM1 on lymphocytes, blocking antitumor immunity [107, 108]. *Streptococcus mutans* colonization in OSCC induces kynurenic acid (KYNA) production, driving CD8^+^ T-cell exhaustion, thereby promoting tumor progression and resistance to PD-L1/IL-1β blockade [109]. In adrenocortical carcinoma, ITM reduces activated CD4^+^ T cells and natural killer T (NKT) cells and CD8^+^ T-cell activity [110]. Microbial metabolites further reinforce this immunosuppression through both systemic and localized pathways. Systemically, via the gut-systemic or respiratory axis, gut-derived SCFAs, including butyrate and propionate, increase Treg ratios and reduce CTLA4 blockade efficacy [111]. Respiratory SCFAs suppress IFN-γ production by T cells and induce exhaustion [112]. Locally within the TME, intratumoral *F. nucleatum*-derived formate activates AhR signaling and promotes Th17 responses [113]. In CRC, tumor-resident *E. coli* enhances lactate production, inducing RIG-I lactylation and impairing CD8^+^ T-cell activity [114]. Beyond adaptive immunity, ITM also shapes the innate immune system that sustains a tumor-promoting niche. Pattern-recognition receptor signaling is central to this process. *F. nucleatum* accumulates in tumor myeloid cells and drives IL-6/pSTAT3/c-MYC signaling via TLR4, fostering M2-like macrophage polarization in CRC [115, 116]. Pancreatic cancer (PC) microbiota differentially activate TLRs to induce immunosuppressive M1 macrophage differentiation and T-cell anergy [28]. *Candida tropicalis* enhances glycolysis in MDSCs, activates NLRP3, and promotes colon tumor formation [117]. Tryptophan metabolites from *Lactobacillus* activate macrophage AhR and promote immunosuppressive tumor-associated macrophages (TAMs) [118]. At the same time, pro-inflammatory signaling further drives tumor progression through both ITM–TME interactions and gut-derived microbial signals. Within the TME, ITM activates NF-κB and related pathways through pattern recognition receptors [119]. ETBF toxin induces a STAT3-NF-κB cascade driving IL-17 and IL-23 release [120], while microbial lipopolysaccharide (LPS) activates TLR4 to recruit macrophages, stimulate IL-1β production, and expand Th17 cells in a reinforcing loop [121, 122].

To stabilize this supportive niche, ITM promotes angiogenesis, biofilm formation, and drug resistance through distinct spatial interactions. Locally, *Rhodococcus* sp.* B513* and *Prevotella intermedia* (*P. intermedia*) enhance vascular endothelial growth factor expression, fostering angiogenesis [123, 124]. Biofilm formation enables microbes to evade host immunity and persist within tumors. Fungal biofilms typically develop through adhesion, hyphal growth, and matrix production, and often incorporat β-glucans and infiltrating neutrophils, indicating active host involvement [125]. *Candida albicans* (*C. albicans*) biofilms support anaerobes such as *C. perfringens* and *B. fragilis*, whose peptidoglycans enhance hyphae and invasion [126]. Other fungi, including *Malassezia*, *Cryptococcus*, and *Aspergillus*, also form biofilms [127–129]. In intestinal tumors, fungal-bacterial consortia have been observed, with metagenomic analysis implicating D-arginine, D-ornithine, and butyrate metabolism in CRC progression [128, 130]. Together, these biofilms and polymicrobial interactions sustain microbial dysbiosis, drive local inflammation, and promote tumorigenesis. Importantly, ITM can also directly reduce the efficacy of anticancer therapy through microbial drug metabolism. In PDAC, Gammaproteobacteria expressing cytidine deaminase long isoform (CDD_L_) convert gemcitabine (GEM) into its inactive form, resulting in chemoresistance [131]. *E. coli* degrades 5-FU through the preTA operon, reducing cytotoxicity, while co-colonization with *F. nucleatum* enhances chemoresistance [132]. Overall, these findings suggest that the indirect pro-tumor effects of ITM are not limited to immune modulation, but also involve broader remodeling of the TME into a niche that supports tumor progression, persistence, and treatment resistance.

### Anti-tumorigenic functions of intratumoral microbiota

#### Restraining tumor metabolism

ITM can suppress tumor progression by rewiring key metabolic pathways in cancer cells, including carbohydrate, amino acid, and lipid metabolism. In CRC, *Streptococcus thermophilus* suppresses glycolysis and counteracts the Warburg effect through β-galactosidase-mediated galactose production and subsequent HK2 downregulation [133]. Similarly, Lachnospiraceae and its metabolite butyrate inhibit the Warburg effect and limit CRC cell proliferation [134]. In intrahepatic cholangiocarcinoma (ICC), intratumoral *Paraburkholderia fungorum* suppresses tumor growth by perturbing alanine, aspartate, and glutamate metabolism [135]. In PDAC, localized *Clostridium butyricum* (*C. butyricum*) and its metabolite butyrate reprogram lipid metabolism by enhancing fatty acid uptake while impairing lipolysis, thereby promoting intracellular lipid accumulation and restricting tumor progression [136]. Such metabolic restraint limits tumor proliferation and may further support the integration of metabolic therapy with immunotherapy by alleviating metabolism-driven immunosuppression.

#### Inducing cell death

Microbial metabolites shape tumor cell fate through diverse cell death modalities. In endometrial cancer (EC), butyrate-producing bacteria, including *Butyrivibrio*, *Clostridium*, and *Faecalibacterium*, enhance progesterone sensitivity by promoting ferroptosis via CISD1 inhibition, leading to iron accumulation and ROS generation [137]. *C. butyricum* and its metabolite butyrate sensitize PDAC cells to ferroptosis by promoting intracellular lipid accumulation through enhanced fatty acid uptake and impaired lipolysis [136, 138]. Butyrate induces lysosome Fe^2^⁺ accumulation and SLC7A11 ubiquitination-mediated degradation in LC stem cells, triggering lysosome-dependent ferroptosis [139]. Propionate suppresses cancer progression by activating ACSL4-mediated ferroptosis [140]. Trimethylamine N-oxide, produced by Clostridiales, triggers GSDME-mediated pyroptosis through PERK activation [141, 142]. By engaging non-apoptotic death programs, ITM and its metabolites emerge as promising candidates for the development of novel tumoricidal agents and for interventions designed to overcome therapeutic resistance.

#### Activating tumor-suppressive signaling

In situ, ITM directly interferes with oncogenic cascades. For instance, tumor-colonizing *E. coli Nissle* 1917 (EcN) promotes apoptosis in CRC by upregulating PTEN and suppressing AKT1 signaling [143]. In contrast, via the gut-systemic axis, gut-resident microbes exert tumor-suppressive effects distally through circulating metabolites. For example, gut-colonizing *Carnobacterium maltaromaticum* produces 7-dehydrocholesterol, which is subsequently converted into vitamin D by *Faecalibacterium prausnitzii*, thereby activating colonic mucosal vitamin D receptor signaling to restrict tumor progression [144]. Furthermore, circulating microbial metabolites like sodium butyrate act systematically as histone deacetylase inhibitors, inducing histone hyperacetylation and p21WAF1/Cip1 expression, which activates Chk1/Chk2-mediated DNA damage responses and selective cancer cell death [145]. These distal anti-tumor effects mediated by gut microbes further expand the boundary of ITM research, and provide theoretical support for the systemic anti-tumor application of oral microbial preparations.

#### Fostering an anti-tumorigenic environment

Microbiota profoundly enhances antitumor immunity and ICB efficacy through two strictly distinct spatial dimensions: systemic priming via the gut-immune axis and tumor-site immune reprogramming.

From a distance, gut-resident microbes and their circulating metabolites systemically prime immune cells before or during their recruitment to the tumor. For example, gut-derived Clostridiales produce trimethylamine N-oxide (TMAO), which systemically amplifies CD8^+^ T cell-mediated responses [146]. Similarly, intestinal *Lactobacillus gallinarum* secretes indole-3-carboxylic acid into the circulation, which antagonizes the IDO1/Kynurenine/AhR axis, blocking Treg differentiation to improve anti-PD-1 efficacy [147]. Inosine, produced by intestinal *Bifidobacterium pseudolongum* (*B. pseudolongum*), systemically induces Th1-regulating gene expression in CD4^+^ T cells [148]. Beyond metabolites, gut colonization by *H. hepaticus* initiates distant immune cascades that induce T follicular helper cells and tertiary lymphoid structure maturation, which subsequently activate B and NK cells [149]. Additionally, a high abundance of intestinal Lachnospiraceae is positively correlated with increased systemic tumor-infiltrating lymphocytes (TILs) [134]. Furthermore, *L. reuteri*-derived indole-3-aldehyde (I3A) activates AhR signaling in T cells to promote IFN-γ production [7, 150].

Conversely, microbes residing directly within the TME shape the immune milieu through immediate, localized interactions. Intratumoral accumulation of *Bifidobacterium* and *Akkermansia* potentiates immunotherapy by locally releasing cyclic diadenylate monophosphate (c-di-AMP), which activates the STING pathway in tumor-associated mononuclear phagocytes and stimulates NK cells and dendritic cells (DCs) [151]. In bile tract cancers, tumor-resident *Clostridia* directly downregulate PI3K signaling in the TME, thereby limiting the local recruitment of myeloid-derived suppressor cells (MDSCs) and promoting CD8^+^ T-cell infiltration [152]. In CRC, tumor-colonizing *Lactobacillus intestinalis* binds local receptors to induce CCL5 secretion via the NOD1/NF-κB pathway, strongly promoting intratumoral DC chemotaxis [146]. In PC, local presence of *Saccharopolyspora*, *Pseudoxanthomonas*, and *Streptomyces* recruits CD8^+^ T cells to establish a proinflammatory milieu [153].

Furthermore, intratumoral bacteria provide antigens for molecular mimicry and cross-reactivity. Bacterial peptides expressed within tumors can be presented by local antigen-presenting cells to activate T cell-mediated responses [154]. Specifically, *Bifidobacterium breve* (*B. breve*) and *E. hirae* prophages express tumor-mimicking peptides locally, boosting T-cell cross-reactivity against tumor antigens [155, 156]. Widespread homology between tumor-associated antigens and peptides from intratumoral Firmicutes and Bacteroidetes further enables local antibacterial CD8^+^ T cells to recognize and attack tumor cells [157]. Finally, localized interventions, such as intratumoral *Clostridium butyricum* MIYAIRI 588 (CBM588), directly induce neutrophil TRAIL release via the TLR2/4-MMP-8 axis within the TME, triggering localized tumor cell apoptosis [158]. Across these studies, a consistent picture emerges that microbiota promotes antitumor immunity through two complementary spatial modes: systemic immune priming outside the tumor and localized immune reprogramming within the TME. Accordingly, both microbial composition and the spatial context of host–microbe interaction should be considered when predicting or harnessing microbiota-dependent therapy benefit.

## Diversity of intratumoral microbiota across cancer types

The ITM is not a monolithic entity, exhibiting substantial heterogeneity in composition, diversity and abundance across organ systems, histological subtypes, disease stages and patient-specific factors. In this section, we systematically catalog the diversity of ITM across human cancers (Table 1), and further summarize the broader patterns that extend across cancer types, including pro-tumorigenic and anti-tumorigenic taxa, and cancer-specific ITM signatures.

**Table 1:** Diversity of intratumoral microbiota across various cancer types

Cancer type	Microbiota composition	Status	Characteristic	Ref
**Brain cancer**	*Acinetobacter, Neisseria macacae, Enterobacter cloacae*	-	Existed in glioblastoma	[4]
	Fusobacteriaceae, Aerococcaceae, *Tissierellaceae*	↑	Predominated in adrenocorticotropic hormone-secreting pituitary neuroendocrine tumors	[159]
	Corynebacteriaceae, S24-7, Aerococcaceae, Clostridiales, F16	↑	Predominated in growth hormone-secreting pituitary neuroendocrine tumors	[159]
**Nasopharyngeal carcinoma**	Epstein-Barr virus	**↑**	Considered a major pathogenic factor for nasopharyngeal carcinoma	[160]
	*Corynebacterium*, *Staphylococcus*	**↑**	Correlated inversely with tumor-infiltrating T lymphocytes and predicted poor clinical outcomes	[35]
	*Fusobacterium nucleatum*, *Prevotella*	**↑**	Exhibited a dose-dependent association with Epstein-Barr virus load	[161, 162]
	*Pseudomonas*, *Cutibacterium*,* Finegoldia*	-	Served as potential nasopharyngeal carcinoma biomarkers	[163]
**Thyroid cancer**	Proteobacteria, Actinobacteria	↑	*-*	[164–167]
	*Sphingomonas*	↑	Correlated positively with lymph node metastasis, served as a prognostic indicator, and distinguished tumor from peritumoral tissues with *Comamonas*	[165–167]
	*Candida albicans*	↑	Associated with advanced tumor–node–metastasis staging	[166, 167]
	*Pseudomonas*,* Rhodococcus*	↑	Predominant in early-stage (tumor stage 1/2) papillary thyroid carcinoma	[165]
	*Streptococcus*,* Granulicatella*	↑	Predominant in advanced-stage (tumor stage 3/4) papillary thyroid carcinoma	[165]
**Oral cancer**	*Porphyromonas gingivalis*	↑	Associated with oral carcinogenesis	[168–170]
	*Fusobacterium nucleatum*,* Prevotella intermedia*	↑	Promoted cancer-related inflammation and served as potential diagnostic or therapeutic targets	[171]
	*Streptococcus*	↓	-	[172]
	*Atopobium*, *Capnocytophaga, Alloprevotella*,* Campylobacter concisus*	↑	-	[173–175]
	*Eubacterium infirmum*, *Actinobaculum*,* Selenomonas*	-	Associated with non-response to neoadjuvant immunotherapy in oral squamous cell carcinoma	[176]
	*Fusobacterium periodonticum*, *Parvimonas micra*,* Streptococcus constellatus*	↑	Associated with advanced oral squamous cell carcinoma stages	[177, 178]
	*Candida albicans*	↑	Exhibited enhanced biofilm formation, hydrolytic enzyme activity, and alcohol metabolism to acetaldehyde, thereby promoting tumorigenesis	[179–181]
	Human papillomavirus	-	Considered a major pathogenic factor for oral cancer	[182]
	Epstein-Barr virus, herpes simplex virus type 1	-	Associated with oral carcinogenesis	[183, 184]
**Lung cancer**	*Corynebacterium*,* Klebsiella pneumoniae*	-	*-*	[4]
	*Escherichia-Shigella*, *Faecalibacterium*,* Pseudomonas*	-	*-*	[185]
	Proteobacteria, Firmicutes, *Blastomyces*, *Aspergillus sydowii*	↑	*-*	[6, 24, 186]
	Actinobacteria, *Halomonas*	↓	*-*	[186, 187]
	*Klebsiella*, *Anaerococcus*,* Enterobacter*	↑	Predominant in lung squamous cell carcinoma	[188]
	*Acinetobacter*, *Pseudomonas*, *Staphylococcus*, *Cyanobacteria*	↑	Predominant in lung adenocarcinoma	[188]
	*Alternaria arborescens*	↑	Predominant in non-small cell lung cancer	[189]
	*Thermus*	↑	Predominant in advanced-stage non-malignant tissues	[190]
	*Aspergillus*, *Agaricus*, *Acidovorax*	↑	Predominant in smoking-associated lung cancer	[6, 191]
	*Veillonella*, *Megasphaera*,* Roseburia*	-	Served as diagnostic biomarkers	[192, 193]
	Bacilli, Lactobacillales, *Streptococcus*	-	Served as a predictor of cancer incidence in asymptomatic patients	[194]
	Pseudomonadales, Actinomycetales	-	Correlated negatively with longer disease-free survival	[195]
**Breast cancer**	*Corynebacterium, Acinetobacter, Fusobacterium nucleatum, Staphylococcus aureus*	↑	-	[4, 6]
	Capnodiales, *Malassezia arunalokei, Aspergillus, Sporobolomyces roseus*	↑	-	[4, 6]
	*Brevundimonas*	↑	Predominant in Luminal A tumors and linked to nucleotide and amino acid metabolism	[196]
	*Mobiluncus*	↑	Predominant in Luminal B tumors, associated with lipopolysaccharide biosynthesis and epithelial invasion pathways	[196]
	*Acinetobacter, Pseudomonas*	↑	Predominant in non-Hispanic White women	[196]
	*Prevotella, Ralstonia*	↑	Predominant in non-Hispanic Black women	[196]
	*Chlamydia, Streptococcus, Dyadobacter*	↑	Predominant in human epidermal growth factor receptor 2 positive breast cancer	[4, 197, 198]
	Proteobacteria, *Corynebacterium*	↑	Predominant in estrogen receptor-positive breast cancer	[4, 197]
	*Streptococcus, Bacillus, Phenylobacterium*	↑	Predominant in triple-negative breast cancer	[4, 196, 197, 199]
	*Yersinia, Azorhizobium*	↑	Predominant in triple-positive breast cancer	[4, 196, 197, 199]
	*Acinetobacter, Bacteroides*	↑	Predominant in tumors with lymph node involvement	[200]
	*Achromobacter*	↑	Predominant in node-negative tissues	[200]
	Thermi*, Hyphomicrobium*	↑	Predominant in stage I tumor	[196, 201, 202]
	*Fusobacterium, Agrococcus*	↑	Predominant in stage III tumor	[196, 201, 202]
	*Propionicimonas*, Micrococcaceae, Caulobacteraceae, Rhodobacteraceae, Nocardioidaceae, Methylobacteriaceae	↑	Predominant in malignant tissues	[202]
**Esophageal cancer**	*Fusobacteria*	↑	Correlated negatively with patient survival	[203]
	*Porphyromonas gingivalis*	↑	Associated with poor differentiation, lymph node metastasis, and advanced tumor–node–metastasis staging	[204]
	*Prevotella*	↑	Predominant in patients with lymph node metastasis and poor prognosis	[205, 206]
	*Staphylococcus, Lactobacillus, Bifidobacterium, Streptococcus*	↑	Produced lactic acid; predominant in esophageal adenocarcinoma	[207]
	*Candida albicans, Cyberlindnera jadinii, Malassezia pachydermatis*	↑	-	[24]
	*Rhodotorula toruloides, Malassezia dermatis, Hanseniaspora lachancei, Spegazzinia tessarthra*	↑	-	[208]
**Gastric cancer**	*Helicobacter pylori*	↑	Associated with intestinal-type gastric cancer	[22, 209]
	*Streptococcus anginosus, Fusobacterium nucleatum, Peptostreptococcus, Prevotella*	↑	Associated with an immunosuppressive tumor microenvironment	[210, 211]
	*Fusobacterium nucleatum*	↑	Predominant in gastric cancer with lymph node metastasis and poor prognosis	[212]
	*Streptococcus anginosus*	↑	Promoted tumor proliferation and metastasis, inhibited CD8 + T-cell infiltration, and remodeled the tumor immune microenvironment via its arginine metabolic pathway by increasing ornithine levels	[213]
	*Candida albicans, Candida tropicalis*	↑	Activated pro-inflammatory pathways	[24, 214]
	Epstein-Barr virus	-	Promoted carcinogenesis through programmed death-ligand 1 upregulation, p53 pathway disruption, and chromatin remodeling	[215–217]
	*Lactobacillus, Acinetobacter, Peptostreptococcus*	-	Distinguished gastric cancer from superficial gastritis	[218]
**Pancreatic cancer**	Enterobacteriaceae, Pseudomonadaceae	↑	*-*	[131]
	*Fusobacterium nucleatum, Exiguobacterium, Pseudomonas, Acinetobacter, Enterococcus, Streptococcus, Helicobacter, Sphingomonas*	-	*-*	[219]
	*Malassezia, Alternaria, Yarrowia*	↑	-	[6, 82]
	Hepatitis B virus, hepatitis C virus	↑	Associated with pancreatic cancer	[220]
	*Acinetobacter, Pseudomonas, Sphingopyxis*	↑	Predominant in basal-like subtype; linked to inflammation and cancer-related signaling pathways	[221]
	*Bradyrhizobium*	↑	Predominant in pancreatic head cancer	[222]
	*Shuttleworthia, Bacillus, Bifidobacterium*	↓	Reduced in pancreatic body/tail cancer	[222]
	*Bifidobacterium pseudolongum*	-	Promoted proliferation and immune evasion through TLR/MyD88/NF-κB activation	[28]
	*Bacteroides, Lactobacillus*	-	Associated with reduced CD4 +, CD8 +, and CD45RO + T-cell infiltration and poor prognosis	[223]
	Gammaproteobacteria	-	Conferred chemoresistance by expressing cytidine deaminase, which metabolizes and inactivates gemcitabine	[131]
	*Malassezia*	-	Promoted tumor progression by binding mannose-binding lectin and activating the complement cascade	[82]
	*Pseudoxanthomonas, Saccharopolyspora, Streptomyces*	↑	Predominant in long-term survivors	[153]
**Liver cancer**	Proteobacteria, Firmicutes, Actinobacteria, Enterobacteriaceae, *Neisseria, Fusobacterium, Helicobacter*	↑	-	[224, 225]
	Fusobacteria, Verrucomicrobia, Staphylococcaceae, Comamonadaceae, *Acidovorax, Staphylococcus*	↑	Predominant in intrahepatic cholangiocarcinoma	[135]
	*Fusobacterium, Neisseria,* Enterobacteriaceae	↑	Predominant in hepatocellular carcinoma	[225]
	Caulobacteraceae, Rickettsiaceae	↓	Reduced in combined hepatocellular carcinoma and intrahepatic cholangiocarcinoma	[226]
	*Pseudomonas*	↑	Predominant in long-term survivors	[226]
	*Malassezia*	-	Downregulated CYP7A1 and CYP27A1 to suppress bile acid synthesis and promoted tumor progression	[227]
	*Enterococcus faecalis, Streptococcus anginosus*	↑	Promoted epithelial-mesenchymal transition and fostered an immunosuppressive tumor microenvironment	[228]
	*Brevibacillus parabrevis*	-	Prevented NK cell ferroptosis and enhanced antitumor immunity by modulating post-translational modifications	[229]
**Colorectal cancer**	*Fusobacterium nucleatum*	↑	Promoted carcinogenesis through immunomodulation, virulence factors (FadA and Fap2), microRNAs, and metabolic alterations; increased with advancing stage; served as an early detection and poor prognosis biomarker	[33, 34, 94, 230–235]
	*Escherichia coli*	-	Produced colibactin, a genotoxin that induces DNA double-strand breaks in host cells	[236, 237]
	*Enterotoxigenic Bacteroides fragilis*	-	Promoted mucosal inflammation and carcinogenesis via IL-17 induction	[238, 239]
	*Clostridium difficile, Streptococcus gallolyticus, Peptostreptococcus stomatis, Prevotella intermedia, Solobacterium moorei*	↑	-	[230, 240–243]
	*Candida albicans, Saccharomyces cerevisiae, Malassezia, Rhodotorula, Pisolithus*	↑	-	[6, 24, 244]
	Human papillomavirus, Epstein-Barr virus, John Cunningham virus, Orthobunyavirus, Inovirus, Tunalikevirus	↑	-	[245–248]
	*Veillonella parvula, Haemophilus, Veillonella*	↑	Predominated in right-sided colon cancer	[230, 249]
	*Streptococcus anginosus*	↑	Predominated in left-sided colon cancer	[230, 249]
	*Peptostreptococcus anaerobius, Bifidobacterium, Ruminococcus*	↑	Predominated in rectal cancer	[230, 249]
	*Fusobacterium hwasookii, Porphyromonas gingivalis*	↑	Predominated in consensus molecular subtype 1 tumors	[250]
	*Selenomonas, Prevotella*	↑	Predominated in consensus molecular subtype 2 tumors	[250]
	*Bacteroides, Ruminococcus, Peptostreptococcus*	-	Associated with KRAS mutation	[251]
	Leptotrichia, Oribacterium, Gallionella	-	Linked to microsatellite instability high colorectal cancer	[251]
**Ovarian cancer**	*Shewanella, Pediococcus, Mycoplasma, Chlamydia, Salmonella*	↑	-	[252–254]
	*Malassezia, Cladosporium, Pneumocystis, Aspergillus clavatophorus*	↑	-	[6, 252]
	Human papillomavirus, Hepadnaviridae, Papillomaviridae, Monkeypox virus, Yaba monkey tumor virus	↑	-	[252]
	*Leishmania, Trichuris*	↑	-	[252]
	*Acinetobacter seifertii*	-	Inhibited M1 macrophage migration	[255]
	*Simiduia, Brachymonas*	-	Served as favorable prognostic markers	[256]
	*Mitsuokella, Salinisphaera*	-	Linked to poor outcomes	[256]
**Prostate cancer**	*Pseudomonas, Faecalibacterium, Cutibacterium, Bacteroides*	↑	-	[257]
	Human papillomavirus-18, Cytomegalovirus, Kaposi’s sarcoma–associated herpesvirus, Epstein-Barr virus, BK virus, John Cunningham virus	-	-	[258]
	*Alternaria, Malassezia, Aspergillus*	-	-	[258]
	*Plasmodium, Trichinella, Sarcocystis*	-	-	[258]
**Endometrial cancer**	*Bacteroides, Faecalibacterium*	↑	-	[259]
	*Anaerococcus, Peptoniphilus, Prevotella*	↑	-	[260]
	*Butyrivibrio, Clostridium, Faecalibacterium*	↑	Predominated in progesterone-sensitive patients	[137]
	*Marinobacterium, Nitrobacter, Mycetocola, Zobellia, Ottowia, Leifsonia, Cyclobacterium, Nitriliruptor, Saccharicrinis*	-	Served as prognostic markers	[261]
**Cervical cancer**	*Lactobacillus*	↓	Fermented glycogen into lactic acid, maintaining acidity, and secreted hydrogen peroxide, bacteriocins, and biosurfactants to inhibit pathogens; its extracellular polysaccharides and peptidoglycans suppressed cancer cell proliferation and induced apoptosis	[262–264]
	*Gardnerella vaginalis, Prevotella bivia, Sneathia*	↑	-	[262, 265]
	*Streptococcus, Fusobacterium, Pseudomonas, Anaerococcus, Acinetobacter*	-	Served as potential biomarkers for distinguishing cervical cancer from healthy individuals	[262]
	Comamonadaceae genus75	↓	Reduced in endocervical adenocarcinoma	[266]
	Micrococcus, Streptococcaceae	↑	Predominated in endocervical adenocarcinoma	[266]
	*Varibaculum, Bosea, Actinotignum, Propionimicrobium*	↑	Predominated in patients receiving anticancer therapy	[267, 268]
	*Atopobium, Aerococcus*	↓	Reduced in patients receiving anticancer therapy	[267, 268]
**Bladder cancer**	*Akkermansia, Bacteroides, Clostridium *sensu stricto*, Klebsiella, Enterobacter*	↑	-	[269]
	*Ralstonia, Sphingomonas*	↑	Predominated in muscle-invasive bladder cancer	[270]
	*Acinetobacter, Staphylococcus, Anoxybacillus_A*	↑	Predominated in non-muscle-invasive bladder cancer	[270]
	*Lactobacillus gasseri, johnsonii*	↑	Predominated in responders to intravesical Bacillus Calmette–Guérin	[271]
	*Corynebacterium kroppenstedtii, Streptococcus*	↑	Predominated in non-responders to intravesical Bacillus Calmette–Guérin	[271]
	*Sphingomonas, Corynebacterium, Capnocytophaga*	↑	Predominated in primary bladder cancer	[272]
	Firmicutes*, Aeromonas, Cupriavidus, Bradyrhizobium*	↑	Predominated in recurrent bladder cancer	[272]
	*Syntrophobotulus, Granulicatella, Xanthomonas, Aquificae, Niabella, Pseudoalteromonas*	-	Correlated with poor outcome and distinct immune infiltration patterns	[273]
**Renal cancer**	Proteobacteria, Firmicutes, Bacteroidetes, Actinobacteria	↑	-	[274–276]
	*Staphylococcus, Corynebacterium, Cutibacterium, Sphingomonas*	↑	-	[276, 277]
	*Plasmodium, Babesia, Toxoplasma, Cytobacillus, Alicyclobacillus, Verrucomicrobium*	-	Linked to favorable outcomes	[278]
	*Colletotrichum, Leuconostoc, Gluconobacter, Parabacteroides*	-	Linked to poor outcomes	[278]
	*Cutibacterium*, *Corynebacterium*, *Clavibacter*, *Simplicispira*	↑	Predominated in papillary carcinoma	[276]
	*Lactococcus, Novosphingobium, Cutibacterium, Psychrobacter*	↑	Predominated in chromophobe carcinoma	[276]
	Epstein-Barr virus, Adenovirus	-	-	[279]
	*Candida, Aspergillus*	↑	-	[279, 280]
**Melanoma**	*Acinetobacter, Actinomyces, Corynebacterium, Enterobacter, Streptococcus*	-	-	[4, 154, 281]
	*Paracoccus marcusii, Staphylococcus aureus*	↑	-	[4]
	*Fusobacterium, Trueperella*	↑	Correlated with poor prognosis	[282]
	*Clostridium*	↑	Predominated in immunotherapy responders	[4]
	*Gardnerella vaginalis*	↑	Predominated in immunotherapy non-responders	[4]
**Non-melanoma skin cancer**	*Staphylococcus aureus*	↑	-	[283]
	*Malassezia, Cutibacterium, Propionibacterium*	↓	-	[284]
	β-human papillomavirus	-	Associated with squamous cell carcinoma; promoted p53 pathway mutations through expression of E6 and E7 oncoproteins under ultraviolet irradiation	[285, 286]
**Hematological malignancy**	Carlavirus, Muromegalovirus, Mamastrovirus, *Thermoanaerobacterium, Labilithrix, Trueperella*	-	Existed in diffuse large B-cell lymphoma tissues	[287]
	*Helicobacter pylori*	-	Associated with gastric mucosa-associated lymphoid tissue lymphoma	[288]
	*Borrelia burgdorferi*	-	Associated with B-cell non-Hodgkin lymphoma	[289, 290]
	*Chlamydia psittaci*	-	Associated with mucosa-associated lymphoid tissue lymphoma at non-gastrointestinal sites	[289, 290]
	Burkholderiaceae, Bacillaceae, Microbacteriaceae	↑	Predominated in bronchus-associated lymphoid tissue lymphoma	[291]
	Human endogenous retrovirus	↑	Predominated in chronic lymphocytic leukemia	[5]
	Epstein-Barr virus	-	Existed in Burkitt lymphoma, Hodgkin lymphoma, and certain natural killer and T-cell lymphomas	[292]
**Bone cancer**	*Sphingomonas yanoikuyae, Actinomyces massiliense, Pseudomonas argentinensis, Enterobacter asburiae*	-	-	[4]
	*Alternaria (other), Aspergillus glabripes, Candida parapsilosis*	-	-	[6]

### Brain cancer

Despite the blood–brain barrier, recent studies demonstrate the existence of ITM in GBM and pituitary neuroendocrine tumors (PitNETs) [4, 159, 293]. Nejman et al. identified enriched *Acinetobacter*, *Neisseria macacae*, and *Enterobacter cloacae* in GBM tissues [4]. Ye et al. further revealed distinct microbial signatures across PitNET subtypes. Specifically, adrenocorticotropic hormone-secreting PitNET was enriched with Fusobacteriaceae, *Tissierellaceae*, and Aerococcaceae, whereas growth hormone-secreting PitNET showed increased abundance of Corynebacteriaceae, S24-7, Aerococcaceae, Clostridiales, and F16 [159]. Given the low-biomass nature of these tumors and the constraints imposed by the blood–brain barrier, future work should focus on robust validation and on determining whether these microbes are persistent residents or context-dependent colonizers.

### Nasopharyngeal carcinoma

NPC is strongly associated with EBV, with the highest incidence observed in East and Southeast Asia [160]. Tumor tissues are enriched with *Corynebacterium* and *Staphylococcus*, where higher bacterial load correlates with reduced T-cell infiltration and poor prognosis [35]. Oral microorganisms may translocate to NPC tumor tissues and promote tumor progression. Among them, *F. nucleatum* and *Prevotella* are consistently enriched in NPC tissues, and exhibit a dose-dependent association with EBV load.[162] Moreover, Lu et al. applied machine learning to identify *Pseudomonas*, *Cutibacterium*, and *Finegoldia* as potential NPC biomarkers [163]. However, it remains unclear whether these bacteria merely reflect EBV burden and immune suppression, or whether they functionally cooperate with EBV in promoting tumor progression.

### Thyroid cancer

Thyroid cancer tissues harbor enriched Proteobacteria, Actinobacteria, *Sphingomonas*, and *C. albicans*, whereas *Comamonas* and *Acinetobacter* are more abundant in NATs.[164, 165, 167] *Sphingomonas* correlates with lymph node metastasis, and *C. albicans* with advanced staging [167]. Microbial diversity increases with tumor stage, shifting from *Pseudomonas* and *Rhodococcus* in early papillary carcinoma to *Streptococcus* and *Granulicatella* in advanced disease [165]. Combined microbial signatures, including *Comamonas* and *Sphingomonas*, show predictive value for distinguishing tumor invasion, highlighting potential diagnostic and prognostic relevance [165]. In summary, thyroid cancer-associated microbiota appears to change with tumor progression and invasive behavior, suggesting that it may serve more as a marker of disease state and aggressiveness than as a universal causal driver.

### Oral cancer

The oral cavity hosts > 700 bacterial species, with Actinobacteria, Bacteroidetes, Firmicutes, Fusobacteria, Proteobacteria, TM7, and Spirochaetes predominating [294, 295]. Fungi (e.g., *Candida*, *Malassezia*), protozoa (e.g., *Trichomonas tenax*), archaea (e.g., *Methanobrevibacter oralis*), and viruses (e.g., bacteriophage) are also present [296–300]. In OSCC, bacterial richness and diversity are increased. *Porphyromonas gingivalis* (*P. gingivalis*) is enriched in gingival carcinoma and is generally considered a risk factor, though some studies paradoxically associate it with better survival [168, 301]. *F. nucleatum*, *P. intermedia*, *Atopobium*, *Parvimonas micra*, and *Capnocytophaga* are also enriched in OSCC and linked to inflammation and progression [171, 174, 177, 178]. Fungal enrichment (e.g., *C. albicans*) promotes biofilm formation, hydrolytic enzyme activity, acetaldehyde production, and immune suppression, contributing to carcinogenesis [179–181, 302]. HPV, especially HPV16, is a major etiologic agent in oral cancer, and recent evidence suggests synergy between HPV oncoproteins and YAP in cancer stemness [182, 303]. EBV and herpes simplex virus type 1 (HSV-1) may also contribute [183, 184]. Notably, because the oral cavity is continuously exposed to external microbes, the central challenge is not only to identify enriched taxa, but to distinguish true tumor-associated functional players from background colonizers.

### Lung cancer

The lung microbiome comprises Bacteroidetes, Firmicutes, Proteobacteria, and Actinobacteria, with *Prevotella*, *Streptococcus*, *Neisseria*, and *Fusobacterium* as common genera [304]. LC tissues harbor *Corynebacterium*, *K. pneumoniae*, *Escherichia-Shigella*, *Faecalibacterium*, and *Pseudomonas *[4, 185]. Compared to NATs, tumors show increased Proteobacteria, Firmicutes, *Blastomyces*, and *Aspergillus sydowii *[6, 24, 186], and decreased abundances of Actinobacteria and *Halomonas *[186, 187]. Microbial composition varies by histology: squamous cell carcinoma enriches *Klebsiella* and *Anaerococcus *[188], while adenocarcinoma favors *Acinetobacter* and *Staphylococcus *[188]; advanced non-malignant tissues show increased *Thermus *[190], and smoking-associated tumors are enriched in *Aspergillus*, *Agaricus*, and *Acidovorax *[6, 191]. Importantly, microbiome-based classifiers show diagnostic potential [192, 193], while reduced Pseudomonadales and Actinomycetales are associated with longer disease-free survival [195].

### Breast cancer

BC is the most common malignancy in women worldwide [305]. Although historically regarded as sterile, breast tissues contain diverse microbes, including *Prevotella*, *Streptococcus*, and *Corynebacterium *[306, 307]. BC tissues show greater microbial diversity than NATs, with enrichment of *Acinetobacter*, *F. nucleatum*, *S. aureus*, and *Methylobacterium radiotolerans*, as well as fungi including *Malassezia arunalokei* and *Aspergillus *[4, 6, 201]. Notably, *Methylobacterium radiotolerance* was detected in pathologically negative sentinel lymph nodes, suggesting a potential role in tumor cell migration [308]. Microbial signatures vary by molecular subtype, race, receptor status, stage, and lymph node involvement [4, 196–198, 200–202]. For example, *Brevundimonas* is enriched in Luminal A tumors, *Mobiluncus* in Luminal B, and *Streptococcus* in human epidermal growth factor receptor 2-positive and triple-negative breast cancer subtypes [4, 196–198]. Lymph node involvement correlates with *Acinetobacter* and *Bacteroides *[200]. These microbial signatures hold promise for risk prediction and prognostic stratification.

### Esophageal cancer

The esophageal microbiome is shaped by swallowing and reflux [309]. The distal healthy esophagus is mainly colonized by Firmicutes, Bacteroidetes, and Verrucomicrobia, with *Streptococcus* as the dominant genus [310]. Under pathological conditions, the esophageal microbiota undergoes dysregulation, with Type II microbiota enriched in Gram-negative taxa such as *Campylobacter concisus* and *Escherichia*, which are strongly associated with esophagitis, Barrett’s esophagus, and gastroesophageal reflux disease [311]. Esophageal adenocarcinoma (EAC) and ESCC show reduced α-diversity and enrichment of *Fusobacteria*, *P. gingivalis*, and *Prevotella*, all linked to poor prognosis [203, 204, 206, 312]. Lactic acid-producing bacteria, including *Staphylococcus* and *Lactobacillus*, are prominent in EAC, and may contribute to metabolic reprogramming [207]. Fungal taxa such as *C. albicans* and *Malassezia* are also detected [24]. Overall, the esophageal microbiota may serve as biomarkers, but validation requires large multicenter studies.

### Gastric cancer

GC is classically linked to *H. pylori*, the only bacterium classified as a Group I carcinogen, which remodels the gastric niche, reduces microbial diversity, and induces chronic inflammation [22, 209]. However, only a fraction of infected individuals develop cancer, highlighting the role of a broader microbial ecosystem. High-throughput sequencing has revealed abundant microbial communities in the stomach [313]. *F. nucleatum* promotes metastasis via NF-κB activation [212], while *Streptococcus anginosus* (*S. anginosus*) enhances proliferation and immune evasion through arginine metabolism [213]. Beyond bacteria, fungi and viruses also contribute through pro-inflammatory signaling, PD-L1 upregulation, and chromatin remodeling [214–217]. Microbial composition shifts dynamically along the cascade from gastritis to carcinoma, with reduced diversity and pathogen overgrowth [314]. Based on the distinct microbial differences between tumors and NATs, predictive models have been developed for early GC diagnosis [23, 315]. Microbial signatures have enabled diagnostic classifiers with high accuracy (area under the curve (AUC) > 0.9) [218], and indices such as the microbial dysbiosis index show potential for early detection and risk stratification [316]. Taken together, gastric carcinogenesis is best viewed as a process shaped by the broader gastric microbial ecosystem during disease progression. This may help explain differences in cancer risk among infected individuals and supports the potential value of microbial signatures alongside *H. pylori* status in early detection.

### Pancreatic cancer

PDAC tissues show higher microbial burden and diversity than the normal pancreas, with enrichment of Gammaproteobacteria, *F. nucleatum*, *Pseudomonas*, and *Streptococcus *[219, 317]. Fungal load is markedly increased, with *Malassezia*, *Alternaria*, and *Yarrowia* predominating [6, 82]. In terms of viruses, enrichment of HBV and HCV in PC tissues has been reported [220]. Distinct microbial signatures characterize PDAC subtypes. The basal-like subtype harbors more bacteria, including *Acinetobacter* and *Pseudomonas*, while pancreatic head cancer is enriched in *Bradyrhizobium* and body/tail cancer is depleted of *Shuttleworthia*, *Bacillus*, and *Bifidobacterium *[221, 222]. Significantly, ITM exerts both pro- and anti-tumorigenic influences in PDAC [28, 131, 223]. *B. pseudolongum* promotes immune evasion via TLR/NF-κB signaling [28], while Gammaproteobacteria confer GEM resistance via CDD_L _[131]. Conversely, high α-diversity and specific taxa (e.g., *Pseudoxanthomonas*) correlate with long-term survival and T-cell activation [153].

### Liver cancer

Liver cancer is closely associated with HBV, liver cirrhosis, and alcoholic liver disease [318]. Tumor tissues are enriched in Proteobacteria, Firmicutes, *Fusobacterium*, and *Helicobacter*, with microbial profiles differing by subtype [224, 225]. *Fusobacterium* and Enterobacteriaceae correlate with hepatocellular carcinoma (HCC), while ICC shows higher diversity and enrichment of Staphylococcaceae and *Acidovorax *[135, 225, 226]. *Stenotrophomonas maltophilia* in cirrhotic HCC induces hepatic stellate cell senescence [319], and Streptococcaceae and *Lactococcus* are markers of cirrhotic HCC [320]. Tumor-resident *Malassezia* promotes tumor progression by suppressing bile acid synthesis [227], and *S. anginosus* fosters EMT and immunosuppression [228]. Conversely, *Brevibacillus parabrevis* enhances NK cell activity [229]. Prognostically, *Pseudomonas* enrichment associates with long-term survival [226]. Interestingly, liver cancer may provide a useful setting for studying how local and systemic microbial influences intersect within the same tumor ecosystem, given the liver’s intimate connection to gut-derived and biliary signals.

### Colorectal cancer

The gut microbiota is dominated by Firmicutes, Bacteroidetes, Proteobacteria, Actinobacteria, and Fusobacteria [321], with fungi (e.g., Ascomycota, Basidiomycota) and viruses (e.g., bacteriophages) also present [322–324]. The balanced gut ecosystem becomes markedly dysregulated in CRC, with enrichment of Firmicutes, Bacteroidetes, *F. nucleatum*, colibactin-producing *E. coli*, ETBF, *Clostridium difficile*, and *Streptococcus gallolyticus *[230, 236, 238, 243], alongside fungal (e.g., *C. albicans*, *Malassezia*) and viral (e.g., HPV, EBV) involvement [6, 24, 247]. Microbial patterns vary by tumor site, stage, and molecular subtype [34, 230, 249–251]. Evidence indicates that ITM also promotes CRC metastasis [325]. *E. coli* disrupts the GVB to promote premetastatic niche formation, while *F. nucleatum* drives metastasis through metabolic reprogramming, m6A modification, and exosome production [30, 34, 94, 96, 326]. Microbial biomarkers (e.g., *F. nucleatum* in blood or stool) show diagnostic and prognostic value [233, 234]. Moreover, metabolites such as butyrate and indole-3-lactic acid (ILA) influence immunity and therapy response [327, 328]. At the same time, in CRC, the overlap between luminal microbiota and ITM makes it essential to distinguish local tissue-resident effects from broader gut microbial influences.

### Ovarian cancer

Ovarian tissues harbor Proteobacteria, Firmicutes, Bacteroidetes, Lactobacilli, and HPV [252, 329]. Ovarian cancer (OC) harbors increased diversity compared with normal tissue, with enrichment of *Chlamydia*, *Mycoplasma*, *Shewanella*, and *Salmonella*, as well as fungi (e.g., *Malassezia*, *Cladosporium*), multiple tumor-associated viruses (e.g., HPV, Hepadnaviridae, Papillomaviridae), and protozoa such as *Leishmania* and *Trichuris *[252]. Microbiota influence immunity within the TME: *Acinetobacter seifertii* suppresses M1 macrophage migration, while microbial signatures associate with immune checkpoint expression and therapy response [255, 256]. In terms of prognosis, *Simiduia* and *Brachymonas* correlate with favorable outcomes, whereas *Mitsuokella* predicts poor survival through promoting angiogenesis and proliferation [256].

### Prostate cancer

The prostate microbiome includes *Propionibacterium*, *Streptococcus*, *Corynebacterium*, and *Staphylococcus*, with *Propionibacterium* being the most prevalent and implicated in prostatic inflammation [330, 331]. These microorganisms may originate from the glans, urine, skin, or gut [257, 331]. Prostate cancer (PCa) tissues enrich *Pseudomonas*, *Faecalibacterium*, *Cutibacterium*, and *Bacteroides *[257]. Using an array-based metagenomic and capture-sequencing approach, Banerjee et al. detected viruses (e.g., HPV-18, Cytomegalovirus, KSHV, EBV, BK virus, John Cunningham virus), fungi (e.g., *Alternaria*, *Malassezia*, *Aspergillus*), and parasites (e.g., *Plasmodium*, *Trichinella*, *Sarcocystis*) in PCa tissues [258]. These microorganisms may influence prostate-specific antigen and androgen levels, cancer cell stemness, and tumor metastasis [103, 332]. However, the prostate is readily influenced by adjacent microbial reservoirs, making it essential to clarify microbial origin before assigning mechanistic significance.

### Endometrial cancer

The healthy endometrium is dominated by *Lactobacillus *[333]. EC shows reduced diversity and enrichment of *Bacteroides*, *Faecalibacterium*, *Micrococcus*, *Porphyromonas somerae*, and *Anaerococcus *[259, 334, 335]. Microbial composition influences therapy response: progesterone-sensitive patients harbor higher α- and β-diversity, with enrichment of butyrate producers such as *Butyrivibrio* and *Clostridium *[137]. For prognosis, a risk model based on ten microbial genera effectively stratified high- and low-risk groups, achieving AUC values exceeding 0.6 at 2, 4, 6, and 8 years, demonstrating robust predictive performance for clinical prognosis [261].

### Cervical cancer

The cervicovaginal microbiota is classified into five community state types (CSTs), with *Lactobacillus*-dominated CSTs associated with a healthy cervical environment, whereas anaerobe-enriched CST IV is linked to persistent HPV infection [336]. As HPV progresses to cervical intraepithelial neoplasia and CC, microbial diversity increases and *Lactobacillus* declines, while taxa such as *Gardnerella*, *Prevotella*, and *Sneathia* become dominant [337]. In CC, there is enrichment of *Streptococcus*, *Fusobacterium*, *Pseudomonas*, *Anaerococcus*, and *Sneathia*, accompanied by depletion of Lactobacillus [262, 266]. Functionally, *Lactobacillus* produces lactic acid and bacteriocins, which inhibit pathogens and tumor growth [263, 264]. By contrast, *Sneathia* and *Fannyhessea* promote inflammation and barrier disruption [338, 339], whereas *Pseudomonas* exacerbates progression via exotoxin A [340]. Moreover, clinical interventions (e.g., LEEP, radiotherapy) can further alter microbial composition [267, 268]. These microbial biomarkers also show potential clinical value, as *Streptococcus* and *Fusobacterium* may aid diagnosis and are associated with treatment resistance [262, 341].

### Bladder cancer

BCa shows reduced diversity and enrichment of *Acinetobacter*, *Anaerococcus*, *Rubrobacter*, *Fusobacterium*, and *Actinobaculum* in urine [342], and *Akkermansia*, *Bacteroides*, *Clostridium *sensu stricto, and *Klebsiella* in tissues [269]. Pathological subtype and sex also shape communities. Muscle-invasive bladder cancer enriches *Ralstonia* and *Sphingomonas,* while non-muscle-invasive bladder cancer harbors *Acinetobacter, Staphylococcus,* and *Anoxybacillus_A*.[270] Male cancer tissues show higher diversity, with female urine enriched in *Lactobacillus* and male urine in *Corynebacterium *[343]. Bacillus Calmette–Guérin (BCG) treatment increases *Lactobacillus*, and responders show higher *Lactobacillus gasseri* and johnsonii [271, 344]. Recurrent BCa enriches *Aeromonas* and *Cupriavidus *[272]. Prognostic signature comprising *Syntrophobotulus* and *Granulicatella* correlates with poor outcome [273].

### Renal cancer

Renal tissues include Proteobacteria, Firmicutes, Bacteroidetes, and Cyanobacteria [276, 345]. Renal cell carcinoma (RCC) tissues show increased microbial load but reduced diversity, with enrichment of *Staphylococcus*, *Corynebacterium*, and *Sphingomonas*, while *Klebsiella* and *Streptococcus* are more common in normal tissue [275, 276]. EBV, ADV, and fungi (e.g., *Candida*, *Aspergillus*) are also detected in RCC tissues, and may influence tumor progression and immunotherapeutic responses [279, 280]. Subtype-specific profiles are evident, with papillary carcinoma enriched in *Cutibacterium* and chromophobe carcinoma in *Lactococcus*.[276] Prognostic taxa include parasites (e.g., *Plasmodium*, *Babesia*) linked to favorable outcomes and bacteria such as *Leuconostoc* and *Parabacteroides* associated with poor survival [278]. These findings fill critical gaps in our understanding of renal cancer microbiota, and offer new avenues for sensitizing RCC to immunotherapy via ITM modulation.

### Melanoma

In normal human skin, *Corynebacterium, Propionibacterium*, and *Brevibacterium* are the predominant commensals [346]. Melanoma enriches *Acinetobacter*, *Actinomyces*, *Corynebacterium*, *Enterobacter*, and *Streptococcus *[4, 154, 281], some of which correlate with poor prognosis [282]. Bacteria-derived peptides presented on histocompatibility complex molecules modulate antitumor immunity [154, 347]. *Staphylococcus epidermidis* produces 6-N-hydroxyaminopurine, suppressing melanoma growth [348]. In ICB-responsive melanoma patients, *Clostridium, Eudoraea, and Desulfonatronospira* are enriched and associated with enhanced cytotoxic T-cell activity [4, 349], supporting a link between ITM and immunotherapy response.

### Non-melanoma skin cancer

Non-melanoma skin cancer (NMSC), comprising basal cell carcinoma and squamous cell carcinoma (SCC), is frequently preceded by actinic keratosis [283, 350]. NMSC is enriched in *S. aureus*, while commensals such as *Malassezia* and *Cutibacterium* are depleted, suggesting protective roles [283, 284]. Immunocompromised patients with NMSC are more susceptible to HPV infection [351, 352]. β-HPV, particularly β-HPV38, contributes to SCC by promoting p53 mutations under UV exposure [285, 286]. Mendelian randomization analyses further suggest causal links between microbiota and skin cancer, with *Propionibacterium* and *Staphylococcus* increasing risk, while *Corynebacterium* and *Rhodobacteraceae* appear protective [353]. This ecological perspective may be especially useful for understanding how microbial alterations cooperate with other carcinogenic factors in driving skin cancer development.

### Hematological malignancy

Hematologic malignancies, including leukemia, lymphoma, and multiple myeloma, also harbor ITM [354]. In diffuse large B-cell lymphoma, over 1,400 genera have been detected, with subsets linked to prognosis [287]. *H. pylori* infection drives gastric mucosa-associated lymphoid tissue (MALT) lymphoma, and eradication therapy achieves durable remission [288]. Other microbial associations include *Borrelia burgdorferi* in B-cell non-Hodgkin lymphoma and *Chlamydia psittaci* in non-gastrointestinal MALT lymphoma [289, 290]. Bronchus-associated lymphoid tissue lymphoma enriches Burkholderiaceae, Bacillaceae, and Microbacteriaceae [291]. Viruses are also prominent contributors: EBV is implicated in Burkitt lymphoma and Hodgkin lymphoma, while endogenous retroviruses are activated in chronic lymphocytic leukemia [292]. Collectively, the strongest evidence still comes from a limited number of pathogen-cancer pairs, suggesting that future work should distinguish well-established causal relationships from broader associative microbial signals.

### Bone cancer

Bacteria such as *Sphingomonas yanoikuyae*, *Actinomyces massiliense*, *Pseudomonas argentinensis*, and *Enterobacter asburiae* have been detected [4]. Heymann et al. identified LPS, a Gram-negative bacterial outer membrane component, in osteosarcoma (OS) specimens, suggesting bacterial involvement in the TME [355]. Supporting this, an OS mouse model demonstrated that intratumoral LPS injection enhanced CD8^+^ T lymphocyte infiltration and suppressed tumor growth [356]. Furthermore, fungal colonization has also been observed, with species including *Alternaria* (other), *Aspergillus glabripes*, and *Candida parapsilosis *[6]. Overall, although the microbial landscape of bone cancer remains only sparsely characterized, future studies should prioritize identifying functionally relevant microbial products and validating their local effects on tumor immunity and metastatic behavior within bone tumors.

Taken together, this systematic characterization of ITM across more than 20 human cancer types reshapes the “sterile tumor” dogma and suggests two broad patterns of ITM organization: conserved pan-cancer microbial features and cancer-specific signatures shaped by anatomical niche, oncogenic drivers, and disease trajectory. Across diverse malignancies, *F. nucleatum* emerges as the most widely validated pro-tumorigenic taxon, with consistent enrichment and established links to inflammatory signaling, metabolic rewiring, and epigenetic regulation spanning NPC, OSCC, BC, esophageal cancer, GC, PDAC, liver cancer, CRC, and CC. Other pro-tumorigenic taxa include *P. gingivalis*, *S. anginosus*, and the fungal genus *Malassezia*. In contrast, *Lactobacillus* represents the dominant pan-cancer anti-tumorigenic taxa, mediating protective effects via maintaining mucosal barrier integrity, producing anti-tumor metabolites such as lactic acid and bacteriocins, and limiting pathogenic microbial colonization across esophageal cancer, CC, and BCa. Additional microorganisms with anti-tumor functions include *Butyrivibrio* and *Clostridium*. Beyond these conserved features, ITM composition is also shaped by the local tissue microenvironment: malignancies in microbially exposed organs (e.g., respiratory, oral, and gastrointestinal tracts) exhibit higher microbial biomass and stronger links to commensal microbes, while immune-privileged sites (e.g., brain, kidney, and bone) harbor low-biomass communities limited by technical challenges in contamination control and functional validation. This niche-specific selection underpins cancer-specific microbial signatures, including *H. pylori* in GC, HPV in CC, and EBV in NPC. Despite these transformative insights, critical unresolved gaps remain across the field, including distinguishing tumor-resident microbes from transient background colonizers, and bridging correlative clinical observations with causal mechanistic evidence. Importantly, these pan-cancer and cancer-specific ITM features are not merely diagnostic or prognostic biomarkers, as their ability to remodel the genomic, immune, and metabolic landscape of the TME directly determines anti-cancer therapy efficacy, leading us to next delineate the multifaceted effects of ITM on diverse antitumor therapies.

## Effects of intratumoral microbiota on antitumor therapy

ITM is increasingly recognized as a component of the TME and may influence tumor progression as well as therapeutic response [357]. Although some therapy-associated effects may involve microbes within tumor tissues, direct mechanistic evidence for ITM remains limited, and most established microbiota-mediated effects on treatment response are linked instead to gut microbiota-dependent systemic immune regulation. The following sections summarize how intratumoral and gut microbiota influence the efficacy of radiotherapy (RT), chemotherapy (CT), immune checkpoint inhibitor therapy, adoptive cell transfer therapy (ACT), and CpG oligodeoxynucleotide-based therapy.

### Radiotherapy

RT, a cornerstone of treatment for solid tumors, relies on inducing DNA damage and activating antitumor immune responses [358]. While the role of microbes in chemoresistance has been extensively studied, investigations into how ITM affects RT efficacy are only beginning to emerge.

Current evidence suggests that ITM may directly affect RT efficacy by modulating tumor cell death pathways and radiosensitivity. In NPC, *F. nucleatum* was shown to promote radioresistance by attenuating radiation-induced PANoptosis and preserving mitochondrial homeostasis [161]. These findings support the possibility that defined ITM can reduce radiation responsiveness within the local tumor niche.

In addition to therapeutic efficacy, ITM may also have value as a biomarker of treatment outcome. In locally advanced rectal cancer, intratumoral enrichment of *Ruminococcus* was associated with pathological complete response, whereas increased abundance of *Fusobacterium* and *Porphyromonas* was associated with non-response and resistance [359]. Likewise, persistence of *F. nucleatum* after neoadjuvant chemoradiotherapy has been associated with a higher risk of local recurrence [360].

By contrast, several lines of evidence relevant to RT tolerance and systemic response are currently supported more strongly by studies of the gut microbiome. For example, gut-derived butyrate from Lachnospiraceae can impair radiation efficacy by suppressing STING-dependent type I interferon production and weakening cytotoxic T-cell responses [361, 362]. Conversely, modulation of the gut microbiota through fecal microbiota transplantation (FMT) or probiotics has shown preliminary potential to mitigate collateral radiation-induced toxicity, including gastrointestinal mucositis, and may improve treatment tolerance [363–365].

Although the field is still at an early stage and direct mechanistic evidence remains limited, emerging studies suggest that interactions between microbes and RT may influence both therapeutic efficacy and treatment-related toxicity. Specific ITM features and ITM-immune signatures hold promise as biomarkers of RT response and toxicity. Concurrently, microbiota-targeted interventions show preliminary potential to improve efficacy and reduce toxicity. Further studies are required to elucidate underlying mechanisms and validate clinical translation.

### Chemotherapy

CT remains central to the management of advanced cancers, yet resistance is widespread and undermines efficacy [366]. Emerging evidence also shows that ITM actively shapes chemoresistance by modulating drug activity, host immunity, and tumor cell survival [131].

Some ITM members directly inactivate chemotherapeutic drugs through degradation or modification, significantly reducing efficacy. In PC, CDD_L_-expressing Gammaproteobacteria, including members of Enterobacteriaceae and Pseudomonadaceae, metabolize GEM into an inactive form, thereby reducing treatment efficacy [131]. Separately, Mycoplasma hyorhinis has also been reported to confer gemcitabine resistance [131]. These findings indicate that, in selected tumors, microbial drug inactivation may represent a direct mechanism of chemoresistance within the TME.

Beyond direct drug metabolism, ITM has also been implicated in malignant progression during or after CT. In CRC, *F. nucleatum* has been linked to both chemoresistance and metastatic progression [367]. This raises the possibility that tumor-associated microbes may influence not only immediate therapeutic response but also longer-term outcomes such as postoperative recurrence and distant spread.

Nevertheless, the clinical translation of these findings remains at an early stage. While CT is one of the strongest areas of evidence for direct ITM-associated effects, the extent to which microbial targeting reproducibly improves long-term survival across tumor types remains to be established.

### Immune checkpoint inhibitor therapy

Immune checkpoint inhibitors (ICIs) targeting PD-1, PD-L1, and CTLA-4 have transformed cancer therapy, yet efficacy varies and resistance is common [368]. Growing evidence implicates the microbiome as a determinant of ICI outcomes [369]. While most insights come from the gut, ITM may directly remodel the local immune milieu, though mechanisms remain underexplored [370]. Core microbe-immune pathways identified in the gut may provide a useful framework for investigating ITM-associated mechanisms.

At present, several of the clearest mechanistic insights relevant to ICI therapy continue to come from gut microbiome studies. For example, commensal gut microbes such as *B. pseudolongum* produce inosine, which can enhance the efficacy of anti-CTLA-4 and anti-PD-L1 therapies [148]. Microbial antigens may also mimic tumor-associated epitopes and thereby enhance T-cell recognition and ICI efficacy. For instance, *E. hirae* phage-encoded TMP1 mimics the tumor antigen PSMB4, co-activating CD8^+^ T cells and improving PD-1 efficacy [155], while *Bifidobacterium breve* SVY epitopes resemble the tumor antigen SIY and may facilitate tumor recognition [156].

Beyond these gut-derived mechanisms, emerging evidence also links ITM features to ICI responsiveness. Response-associated species such as *Priestia megaterium* and *Burkholderia cepacia* have been identified in this setting [371]. Consistent with this, intratumoral *F. nucleatum* has, under certain conditions, also been reported to enhance the efficacy of PD-L1 blockade in colorectal cancer [357].

### Adoptive cell transfer therapy

ACT represents a rapidly evolving immunotherapeutic strategy in which autologous or allogeneic immune cells are expanded or genetically engineered ex vivo to enhance antitumor activity before reinfusion into the patient. Traditional ACT approaches primarily include TIL therapy, T-cell receptor-engineered T cells, and chimeric antigen receptor T cells, while emerging platforms extend to NK cells, cytokine-induced killer cells, and macrophage-based therapies [372]. ACT has shown significant efficacy in hematologic malignancies and some solid tumors [373, 374]. Its antitumor mechanism relies on enhancing T cell recognition and cytotoxicity while counteracting the immunosuppressive TME [375].

Compared with RT, CT, and ICI therapy, evidence linking ITM to ACT remains limited. Most available studies instead concern the gut microbiome and its influence on host immunity, antigen presentation, and treatment-associated toxicity. In preclinical settings, modulation of the gut microbiota, including vancomycin-induced dysbiosis, has been shown to alter CAR-T efficacy and, in some models, enhance antitumor activity through improved antigen cross-presentation. However, clinical studies suggest that broad-spectrum antibiotic exposure before anti-CD19 CAR-T therapy is associated with worse survival and increased toxicity [376].

Direct evidence that endogenous ITM modulates ACT remains scarce. Related insights currently come mainly from engineered microbial platforms designed to enhance ACT locally within tumors. For example, probiotic-guided CAR-T cells have been developed as tumor-localized platforms that deliver synthetic targets in situ and have shown safe and effective antitumor activity in preclinical models [377]. However, these approaches should be distinguished from direct manipulation of endogenous ITM. At present, whether naturally occurring ITM influences ACT efficacy, relapse, or toxicity in a comparable manner remains unclear.

### CpG oligodeoxynucleotide-based therapy

CpG oligodeoxynucleotides activate the TLR9 receptor in B cells and plasmacytoid dendritic cells, thereby initiating NF-κB signaling and inducing cytokine and chemokine production [378]. It has potent immunostimulatory activity and potential antitumor effects. The antitumor efficacy of CpG therapy appears to depend, at least in part, on an intact commensal gut microbiota. In antibiotic-treated or germ-free settings, responses to CpG oligonucleotides are markedly impaired. Under these conditions, tumor-infiltrating myeloid cells fail to produce sufficient inflammatory cytokines and reactive oxygen species, resulting in reduced therapeutic efficacy [379]. However, whether ITM exerts comparable local effects remains unclear.

## Clinical applications of intratumoral microbiota in antitumor therapy

**Fig. 4:**
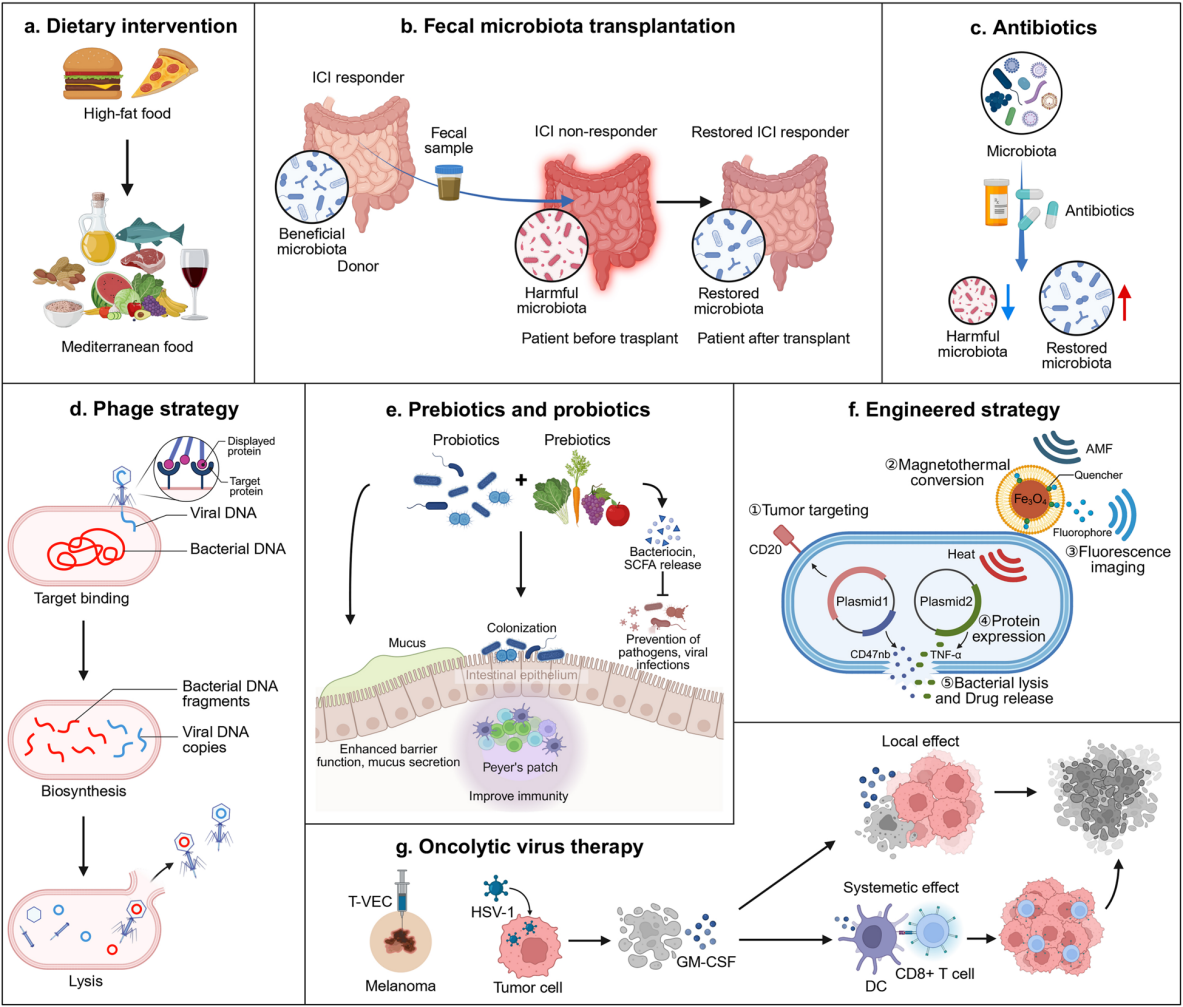
Clinical applications of intratumoral microbiota in antitumor therapy. **a** Dietary intervention. Specific dietary patterns (e.g., Mediterranean diet) reshape microbial composition and metabolite profiles, influencing therapy response, while high-fat diets may promote tumor progression through dysbiosis. **b** Fecal microbiota transplantation. Transfer of fecal microbiota from immunotherapy responders can restore antitumor immunity and overcome resistance in non-responders. **c** Antibiotics. Antibiotics enable the selective elimination of pro-tumorigenic bacteria while preserving beneficial microbiota. **d** Phage therapy. Engineered bacteriophages precisely target and lyse specific intratumoral pathogens, and can be modified to deliver therapeutic payloads. **e** Prebiotics and probiotics. Prebiotics stimulate the growth of beneficial bacteria, while probiotics enhance barrier function, produce antimicrobial compounds, and modulate immune responses. **f** Engineered strategy. Synthetic biology approaches enable the design of bacteria with tumor-targeting capabilities and programmable therapeutic functions. **g** Oncolytic virus therapy. Genetically engineered oncolytic viruses selectively infect and lyse tumor cells, while stimulating antitumor immunity through immunogenic cell death and cytokine induction. Abbreviations: ICI, immune checkpoint inhibitor; SCFA, short-chain fatty acid; T-VEC, talimogene laherparepvec; HSV-1, herpes simplex virus type 1; GM-CSF, granulocyte–macrophage colony-stimulating factor; AMF, alternating magnetic field; TNF-α, tumor necrosis factor alpha; CD20, cluster of differentiation 20; CD47nb, CD47 nanobody; DC, dendritic cell

ITM is increasingly recognized in tumors and may influence tumor progression and therapeutic response by modulating tumor cell function and local immunity [380]. However, the therapeutic approaches discussed in this section differ in both evidential support and mode of action. Selective antibiotics, phage strategy, engineered strategy, and oncolytic virus therapy are intended to act within tumor tissues or directly target ITM, whereas dietary intervention, FMT, and most prebiotic and probiotic strategies are currently supported more strongly by evidence of gut microbiota remodeling and systemic immune modulation. Their direct effects on established ITM remain less well defined. Despite challenges of mechanistic complexity, limited precision, and scarce clinical validation, microbiota modulation is increasingly recognized as a promising adjunct to conventional cancer treatment (Fig. 4, Table 2).

**Table 2:** Clinical trials of intratumoral microbiota in antitumor therapy

Category	Intervention	Combination	Cancer type	NCT	Phase
**Dietary intervention**	Fasting-mimicking diet	Chemotherapy	Non-small cell lung cancer	NCT03700437	N/A
	Potato starch	Dual-immune checkpoint inhibitors	Solid tumor	NCT04552418	1
	Isocaloric high-fiber diet	Immune checkpoint inhibitors	Stage III-IV Melanoma	NCT04645680	2
	High fiber diet	Neoadjuvant treatment	Early-stage triple-negative breast cancer	NCT06610097	N/A
	Additional dietary fiber through psyllium husk	Radiotherapy	Pelvic cancers	NCT04534075	3
**Fecal microbiota transplantation**	Capsulized fecal microbiota transplant	Immune checkpoint inhibitors	Melanoma	NCT03772899	1
	Capsulized fecal microbiota transplant	Immune checkpoint inhibitors	Colorectal cancer	NCT04729322	2
	Capsulized fecal microbiota transplant	Immune checkpoint inhibitors	Non-small cell lung cancer	NCT05008861	1
	Capsulized fecal microbiota transplant	Immune checkpoint inhibitors	Metastatic lung cancer	NCT05502913	2
	Capsulized fecal microbiota transplant	Radiochemotherapy	Colorectal cancer	NCT06931808	4
**Antibiotics**	Metronidazole	Neoadjuvant therapy	Rectal adenocarcinoma	NCT06569368	2
	Metronidazole + oral chlorhexidine	-	Oral cancer	NCT06627270	2
	Metronidazole	Neoadjuvant radiotherapy	Advanced rectal cancer	NCT06793137	2
	Dexamethasone ± metronidazole	Androgen-pathway therapy	Metastatic castration-resistant prostate cancer	NCT06616597	2
**Prebiotics and probiotics**	*Clostridium butyricum MIYAIRI-588*	Immune checkpoint inhibitors	Stage IV or advanced renal cancer	NCT03829111	1
	*Clostridium butyricum MIYAIRI-588*	Immune checkpoint inhibitors + targeted therapy	Advanced or metastatic renal cancer	NCT05122546	1
	Akkermansia probiotics	Immune checkpoint inhibitors	Microsatellite stable/proficient mismatch repair advanced colorectal cancer	NCT06865521	N/A
	*Lactobacillus johnsonii*	Chemotherapy + Immune checkpoint inhibitors	Microsatellite stable/proficient mismatch repair metastatic colorectal cancer	NCT06823323	N/A
	Prebiotic food-enriched diet	Immune checkpoint inhibitors	Immune checkpoint blockade-refractory melanoma	NCT06250335	2
	Prebiotic food-enriched diet	Neoadjuvant immune checkpoint blockade	Melanoma	NCT06548789	2
	Inulin	Immune checkpoint inhibitors	Recurrent/metastatic head and neck squamous cell carcinoma	NCT05821751	N/A
**Engineered strategy**	*Clostridium Novyi-NT*	Immune checkpoint inhibitors	Treatment-refractory solid tumors	NCT03435952	1
	*Clostridium Novyi-NT*	-	Treatment-refractory solid tumors	NCT01924689	1
	*Listeria monocytogenes–based cancer vaccine CRS-207*	-	Advanced solid tumors	NCT00585845	1
	*Attenuated Salmonella bacteria strain VNP20009*	-	Advanced or metastatic cancer	NCT00004988	1
**Oncolytic virus therapy**	Oncolytic vaccinia virus JX-594 (pexastimogene devacirepvec)	-	Hepatocellular carcinoma	NCT00554372	2
	Oncolytic vaccinia virus JX-594 (pexastimogene devacirepvec)	-	Advanced liver cancer after Sorafenib failure	NCT01387555	2
	Oncolytic adenovirus DNX-2401 (tasadenoturev)	-	Recurrent high-grade glioma	NCT03896568	1
	Oncolytic adenovirus DNX-2401 (tasadenoturev)	Immune checkpoint inhibitors	Recurrent glioblastoma/gliosarcoma	NCT02798406	2
	Recombinant herpes simplex virus type 1 vector Nestin34.5v.2	Chemotherapy	Recurrent glioblastoma	NCT03152318	1

### Dietary intervention

Direct evidence that dietary interventions reproducibly remodel established ITM remains limited. Most available studies instead support an indirect route whereby diet alters gut microbial composition and metabolite production, with downstream effects on systemic immunity and the TME [8, 381, 382].

In this context, dietary fiber and microbiota-derived SCFAs, particularly butyrate, have been linked to enhanced antigen presentation and T-cell activation, which may improve responses to ICIs [383, 384]. Likewise, prebiotics such as resistant starch and inulin can enrich bacterial taxa associated with antitumor immunity [385]. Several clinical trials are underway (e.g., NCT04645680, NCT04552418), although current evidence primarily supports gut microbiota-dependent rather than direct intratumoral effects.

A more tumor-localized possibility is suggested in selected settings where dietary substrates may influence the enrichment of specific tumor-associated microbes. For example, *F. nucleatum* appears to depend on glucose and fructose availability for tumor colonization and can promote chemoresistance through autophagy. Restrictive dietary strategies targeting these nutrient dependencies may reduce their enrichment and sensitize tumors to CT [161]. Overall, these findings suggest that dietary intervention may influence the intratumoral niche in selected contexts, but current evidence mainly supports its adjunctive effects through the gut microbiota–immune axis rather than direct reprogramming of established ITM.

### Fecal microbiota transplantation

FMT is being explored in cancer therapy primarily as a strategy to transfer gut microbial communities associated with favorable immune states, rather than as a direct means of editing established ITM [386].

This concept is supported by studies showing downstream effects on systemic immunity and the TME. In melanoma, fecal material from ICI responders improved antitumor responses in recipient models, and clinical studies further showed that FMT combined with anti-PD-1 therapy could alter the gut microbiome and reprogram the TME in a subset of resistant patients [9, 387].

FMT has also been investigated as a supportive approach for immune-related adverse events, particularly refractory ICI-associated colitis, with case reports and case series suggesting restoration of gut microbial composition together with clinical improvement [388]. However, evidence that FMT reproducibly alters the ITM composition or function is limited. In addition, FMT faces practical limitations, including donor screening, batch variability, and potential pathogen transmission, which have motivated the development of more standardized alternatives such as defined microbial consortia and selected bacterial formulations [389, 390].

### Antibiotics

Certain ITM reduces therapeutic efficacy by altering drug metabolism, inducing immunosuppression, and activating oncogenic signaling [131, 235], making their selective elimination an attractive strategy.

Clinical retrospective studies show that cancer patients receiving antibiotics during GEM-based or 5-FU-based CT have significantly longer progression-free survival (PFS) [391]. Animal studies corroborate this: oral metronidazole markedly reduced *F. nucleatum*, suppressing CRC cell proliferation and tumor formation in mice [34]. In BC models, oral or aerosolized antibiotic combinations reduced tumor burden, enhanced paclitaxel sensitivity, and even blocked *F. nucleatum*-induced metastasis [392]. Moreover, nanoformulations of doxycycline or metronidazole achieved efficient tumor penetration and targeted microbial clearance, synergistically inhibiting tumor growth in vivo [393].

However, broad-spectrum antibiotic use can disrupt microbial homeostasis, impair host immunity, and weaken anti-PD-1/PD-L1 therapy. In patients receiving ICIs, antibiotics are associated with higher risks of irAEs and reduced overall survival [394, 395]. In hematologic malignancies, antibiotics may even accelerate leukemia progression [396]. A meta-analysis reported that antibiotic exposure before or during ICI therapy shortened median overall survival by over six months [394]. Another retrospective analysis suggested that early antibiotic administration exerts stronger negative effects, whereas concurrent use during ICI therapy may be relatively safer [397]. Accordingly, research increasingly emphasizes targeted antibiotic delivery to eradicate ITM while minimizing collateral effects on the gut microbiome. Gao et al. developed metronidazole-fluorouridine nanoparticles that release payloads in response to elevated glutathione in the TME, enabling precise *F. nucleatum* clearance and synergistic tumor control. Similarly, bioinspired nanosystems, such as gastric epithelial cell membrane-coated clarithromycin nanoparticles and platelet membrane-coated vancomycin, demonstrate high specificity and potent bactericidal activity against pathogens like *H. pylori* and methicillin-resistant *S. aureus*, showing promise for solid tumor applications [398, 399].

Collectively, antibiotics are evolving from conventional infection control towards precision intratumoral microbial intervention, with future efforts focused on defining tumor-specific targets, optimal timing, and rational combinations with CT and immunotherapy.

### Phage strategy

Given the limitations of antibiotics in biofilm penetration, resistance control, and microbiome preservation, bacteriophages have emerged as precise tools to modulate the ITM and enhance cancer therapy [400–402].

*F. nucleatum*, enriched in CRC, BC, and GC, exemplifies a key oncomicrobe that adheres to tumor cells, activates inflammation, suppresses immunity, and drives CT resistance [66, 235, 403]. Thus, it represents a key target for microbiome-based intervention. Lytic phages specifically targeting *Fusobacterium* penetrate tumor biofilms, lyse bacteria, and reverse pro-tumorigenic effects, thereby enhancing immunotherapy or CT. A chimeric phage vector conjugated to irinotecan-loaded nanoparticles enabled targeted delivery and significantly enhanced antitumor activity [404]. Moreover, bioinorganic hybrids such as M13@Ag selectively eliminated *F. nucleatum* and improved the tumor immune microenvironment (TIME) by blocking MDSC recruitment, thereby potentiating ICIs and chemotherapies [405]. In addition to direct clearance, engineered phages can deliver antigens or immune molecules to modulate immunity. Phage-displayed anti-PD-L1 peptides activated T cells and suppressed tumors [406]. Similarly, M13 phages displaying the human epidermal growth factor receptor 2 antigen have been developed as a therapeutic vaccine for BC, underscoring the potential of phage-based strategies in immunoprevention [407]. Notably, while phage strategy is highly specific, this dependence on bacterial recognition limits its spectrum of application. Phage cocktails, combining multiple phages, are therefore considered effective for broadening bactericidal coverage and improving outcomes [408]. Moreover, careful preclinical evaluation of phage selection, dosage, and administration routes remains essential to reduce inflammatory responses and immune activation.

Overall, bacteriophages offer a highly specific, low-toxicity means of microbiome modulation with potential to synergize with standard therapies, paving the way for personalized microbiome-based interventions in oncology.

### Prebiotics and probiotics

Prebiotic and probiotic interventions in cancer are currently supported mainly by evidence of gut microbiota modulation and systemic immune priming rather than direct remodeling of established ITM. Prebiotics such as inulin and resistant starch can enhance antitumor immune responses in preclinical models, but these effects are largely interpreted through gut microbiota-dependent mechanisms [409].

A more tumor-localized exception has been described for *L. reuteri*, which was shown to translocate to and persist within melanoma, where its tryptophan metabolite I3A promoted CD8^+^ T-cell activity through AhR signaling and enhanced ICB [7]. In addition, *Lactobacillus plantarum* L168 and its metabolite ILA enhance dendritic cell IL12a production and alter chromatin accessibility, strengthening tumor-infiltrating CD8^+^ T cell responses, although direct evidence that these effects represent stable reprogramming of endogenous ITM remains less clear [150].

Despite these promising effects, outcomes vary across strains and tumor types, with some studies reporting negligible benefit, reflecting host-microbe heterogeneity [410]. Tailored interventions, coupled with gene editing, nanotechnology, and imaging technologies, may help optimize probiotic delivery and functionality.

### Engineered strategy

Advances in synthetic biology have enabled engineered bacteria to serve as multifaceted cancer therapeutics, owing to their low toxicity and ability to express prodrug-converting enzymes, release cytotoxic molecules in a controlled manner, modulate immune responses, and target the tumor stroma to enhance antitumor efficacy [411]. *Attenuated S. typhimurium VNP20009* expressing *E. coli*-derived CDD significantly increased intratumoral 5-fluorouracil levels [412]. *Bifidobacterium infantis*-mediated HSV1-TK/GCV system effectively suppressed BCa progression in mice [413]. Moreover, *Clostridium novyi*-NT induced oncolysis in hypoxic tumors exhibited encouraging clinical activity in patients with advanced malignancies [414, 415].

To improve safety and precision, lysis circuits introduced into bacteria allow drug release upon reaching a threshold population density, thereby minimizing systemic toxicity [416]. A thermosensitive memory circuit was designed to enable EcN to sustain nanobody expression against PD-L1 or CTLA-4 following heat activation, alleviating immunosuppression in the TME [417]. Engineered bacteria have also been utilized to construct microrobots for drug delivery, with *E. coli*, *Serratia marcescens*, and *S. typhimurium* propelling biohybrid microswimmers to release therapeutic agents under magnetic control [418, 419].

In terms of immune stimulation, EcN converts ammonia to L-arginine, augments T-cell activity [420], and has been programmed to deliver GM-CSF or checkpoint nanobodies, clearing colorectal adenomas [421]. EcN has also been engineered to express IL-2, L-arginine, and STING agonists to potentiate immune responses [422]. *S. typhimurium* releasing *Vibrio trauma*-derived FlaB promoted an increase in M1-like macrophages via TLR4 signaling, and increased the production of pro-inflammatory cytokines such as IL-1β and TNF-α [423]. Engineered *Listeria* carrying galactosylceramide activated NKT cells and inhibited metastasis [424]. *E. coli* and *S. typhimurium* releasing anti-CD47 nanobodies suppressed tumor growth and elicited CD8^+^ T cell-mediated distal antitumor effects [425]. Bacteria expressing tumor antigens or immune factors elicit memory T cell responses and mediate tumor cell killing [426]. Bacterial membranes coated with nanoparticles increased colonisation and drug delivery efficiency. Under near-infrared irradiation, *E. coli* MG1655 bearing biomineralized gold nanoparticles produced heat to activate TNF expression within the TME [17]. *S. typhimurium* carrying antigen-adsorbing polymers enhanced dendritic cell uptake of tumor antigens and induced systemic immune responses [427].

Considerable efforts have also focused on improving the tumor-targeting capacity of engineered bacteria. Bacteria have been programmed to sense tumor-associated signals such as hypoxia, elevated lactate, and acidic pH, thereby achieving spatially restricted therapeutic expression [421, 428]. Surface engineering approaches have also enhanced bacterial adhesion. EcN displays ice nucleation proteins (INPs) bound to glycosaminoglycans on CRC cells while simultaneously expressing myrosinase to convert dietary glucosinolates into anticancer molecules [429]. Fusion with RGD peptides or anti-CD20 antibodies further improved cancer cell recognition [430, 431], and conjugation with aptamers such as AS1411 enhanced both tumor targeting and immune activation [432]. Additionally, paclitaxel and perfluoroalkanes have been co-encapsulated in bacteria-nanoparticle complexes to treat hypoxic tumors [433]. Engineered bacteria can also degrade tumor stroma by secreting enzymes, exemplified by hyaluronidase-expressing *S. typhimurium*, which enhanced chemotherapeutic drug penetration in pancreatic tumors [434].

Currently, microbes have been developed to both enhance RT, CT, and immunotherapy, and to serve as tumor imaging probes, biosensors, and preventive vaccine vectors. Although most applications remain preclinical, these approaches highlight the promise of synthetic biology to expand microbial cancer therapeutics, with future work needed to optimize safety, dosing, and precision control in humans [435].

### Oncolytic virus therapy

Oncolytic viruses are genetically engineered to selectively infect and lyse cancer cells, exploiting tumor susceptibility to viral replication and releasing antigens that trigger immunogenic cell death and antitumor immunity. They can also inhibit tumor growth by disrupting the vasculature [436]. Representative oncolytic viruses include DNA viruses (e.g., herpes simplex virus, poxvirus) and RNA viruses (e.g., coxsackievirus, measles virus) [437].

Oncolytic viruses act through diverse mechanisms and are being optimized to enhance therapeutic efficacy. JX-594 replicates in cancer cells with EGFR/Ras pathway activation and type I interferon inhibition [436, 438]. Lymphocytic choriomeningitis virus recruits IFN-producing Ly6C^+^ monocytes and promotes the expansion of tumor-specific CD8^+^ T cells [439]. IL-23-expressing oncolytic viruses overcome immunosuppression by increasing T cell infiltration and effector function [440]. Engineering strategies refine targeting and efficacy, with viruses armed to deliver GM-CSF, IL-2, IL-12, IL-18, chemokines, or BiTEs, and delivery systems such as stem cells, extracellular vesicles, and nanotechnology improving tumor selectivity and lytic activity [440–445]. While intratumoral injection remains the main route, systemic administration is limited by immune clearance, driving innovations such as polymer coatings and cell-based shielding [446]. Clinically, approved oncolytic viruses include T-VEC, the first widely recognized agent expressing GM-CSF for melanoma and currently being evaluated in other solid tumors [447]. H101 was approved in 2005 for head and neck and esophageal cancers [448], while HSV-1-based G47Δ was approved for malignant glioma in 2021 [449]. DNX-2401 demonstrated efficacy in 20% of recurrent malignant glioma patients in a phase I trial [450]. Combination therapies enhance efficacy: oncolytic viruses synergize with CT, RT, or immunotherapy [451]. TG4010 plus PD-1/PD-L1 blockade improved outcomes in non-small cell lung cancer [452]. Coxsackievirus A21 combined with pembrolizumab upregulated PD-L1 with good tolerability [142]. Measles virus plus GEM promoted senescent cancer cell lysis [453].

Despite encouraging progress, clinical translation is limited by viral leakage, systemic toxicity, and immune clearance, and overcoming these barriers is key to realizing the full potential of oncolytic virotherapy.

## Methodological challenges in intratumoral microbiota research

Despite growing interest in the therapeutic relevance of ITM, progress in this field depends on how confidently microbial signals can be detected, interpreted, and functionally validated. These challenges stem both from the low microbial biomass of many tumor specimens, which increases susceptibility to contamination and analytical misclassification, and the limitations of current platforms and models. No existing approach can simultaneously provide sensitive detection, spatial localization, and causal functional readout. The following sections discuss these issues from the perspectives of detection, validation, and functional modelling.

### Detection approaches and interpretive limits

A central challenge in ITM research is the low microbial biomass of many tumor specimens [454]. In this setting, microbial signals are often weak and close to the limits of detection of currently available assays, leading to substantial variability across platforms. This problem is compounded by the fact that no single method currently combines high sensitivity, broad taxonomic coverage, functional readout, and spatial localization [455]. As a result, the key issue is often not simply whether a microbe can be detected, but how such signals can be validly interpreted [12].

Among sequencing-based approaches, 16S rRNA amplicon sequencing remains useful for exploratory profiling because it is relatively sensitive, cost-effective, and compatible with small amounts of input material [454]. It is therefore well-suited to initial surveys and cohort-level comparisons. Its interpretive range, however, is narrow: it is largely restricted to bacteria, is susceptible to primer and polymerase chain reaction (PCR) bias, and usually provides limited species-level resolution with little direct functional information [456]. By contrast, shotgun metagenomics offers a broader taxonomic scope, including bacteria, fungi, and viruses, and can, in principle, recover functional gene content [454]. In tumor samples, however, metagenomic libraries are frequently dominated by host-derived reads, which increases sequencing depth requirements, cost, and the risk that residual host sequences will be misclassified as microbial [457]. In low-biomass tumor tissues, these two approaches are best viewed as complementary rather than interchangeable [458]. Moreover, resources such as MicroPhenoDB can assist in the interpretation of sequencing-derived microbial signals by linking candidate microbes to reported disease phenotypes, core genes, and virulence-associated features [459]. However, such associations should be interpreted as supportive annotations rather than direct evidence of intratumoral function.

Metatranscriptomics adds a distinct layer of information by capturing transcribed microbial RNAs, thereby providing stronger evidence that detected microbes are transcriptionally active within the tumor rather than being inferred solely from residual DNA [455, 460]. This distinction is particularly relevant in cancer tissues, where biological activity is likely to matter more than simple detection. The trade-off is technical difficulty: host transcripts often dominate the library, RNA integrity can be limiting, and microbial signal recovery is especially challenging in low-input tissues [455, 460]. Metatranscriptomics is therefore particularly attractive for mechanistic studies, but it is not yet as scalable or robust as DNA-based approaches for routine profiling [461].

Because bulk sequencing disrupts tissue architecture, in situ and imaging-based methods are especially important in this field. Fluorescence in situ hybridization (FISH)-based and RNAscope-based assays can help verify that microbial signals are physically present within tumor sections and can distinguish intratumoral localization from peri-tumoral or handling-related signals [462]. Immunohistochemical detection of bacterial products such as lipopolysaccharide or lipoteichoic acid may provide supporting evidence for bacterial material within tissue, although such assays do not offer taxonomic resolution and should be interpreted cautiously. More broadly, imaging approaches are valuable not because they replace sequencing, but because they anchor sequencing-derived signals in tissue context.

Spatially resolved and single-cell approaches have begun to narrow the gap between detection and interpretation. Spatial transcriptomics and GeoMx digital spatial profiling have shown that intratumoral bacteria are not randomly distributed, but instead occupy organized microniches associated with distinct epithelial and immune states [462]. At single-cell resolution, invasion-adhesion-directed expression sequencing (INVADEseq) enables simultaneous capture of host transcripts and bacterial 16S-derived signals in bacteria-associated cells, whereas computational frameworks such as single-cell analysis of host-microbiome interactions (SAHMI) can recover and denoise microbial signals from single-cell RNA-seq datasets [460, 463]. These methods are powerful because they connect microbial detection to host-cell identity and local transcriptional programmes. Their current limitations are equally clear: they are technically demanding, costly, and not yet optimized for unbiased, high-sensitivity discovery across diverse microbial taxa. For this reason, the most convincing evidence usually comes from orthogonal validation across sequencing, spatial, and imaging modalities rather than from any single platform alone [455].

### Contamination control and orthogonal validation

In low-biomass tumor specimens, false-positive microbial signals remain a central technical problem rather than a peripheral nuisance [464]. Contamination can be introduced during tissue acquisition, surgical handling, sectioning, nucleic acid extraction, library preparation, or sequencing, and can originate from personnel, laboratory surfaces, adjacent high-biomass tissues, or reagent-derived background DNA [454, 464, 465]. When authentic microbial content is sparse, these background signals can equal or exceed the true biological signal. Under these conditions, contamination control is a prerequisite for credible signal interpretation.

Accordingly, studies of the ITM should be designed with contamination awareness from the outset. Appropriate controls include extraction blanks, no-template controls, and environmental controls processed along with tumor specimens [464]. Additional safeguards include randomized batch processing, strict separation of post-amplification workflows, documentation of sample biomass, and sequencing of controls together with biological samples rather than in independent runs [454, 464]. These measures do not eliminate contamination, but they make it measurable and therefore easier to identify and control analytically.

On the computational side, decontamination is now indispensable. Decontam identifies likely contaminants on the basis of their enrichment in low-concentration samples and/or negative controls [456]. Source tracking for Contamination Removal in microBiomes (SCRuB) builds on this approach by modelling shared contamination sources and well-to-well leakage, which is particularly relevant in plate-based processing [466]. Squeegee provides a de novo strategy for identifying likely reagent contaminants even when negative controls are incomplete or unavailable [467]. At the same time, recent re-analyses of large cancer microbiome datasets have highlighted an additional and equally serious problem: host-read misclassification, whereby human sequences are erroneously assigned to microbial reference databases [457]. In this setting, stringent host-read depletion, careful curation of reference databases, and conservative analytical thresholds are not optional refinements but core requirements for credible inference.

### In vivo strategies for functional validation

Associative studies have rapidly expanded the catalogue of microbes detected in tumors, but establishing function in vivo remains substantially more difficult. A major reason is that no single preclinical model can fully balance experimental control, physiological relevance, and translational value. Gnotobiotic and germ-free mouse models remain the most rigorous systems for testing whether defined microbes or consortia can causally influence tumor growth, metastasis, or therapy response, because they provide maximal control over microbial exposure [468, 469]. Their strength, however, is also their limitation: germ-free animals develop in an altered immunological and ecological context and therefore do not fully reproduce the conditions of human tumor ecosystems.

Antibiotic depletion followed by microbial reconstitution is more experimentally accessible and avoids the developmental alterations inherent to germ-free models, but it introduces its own confounders [468, 470]. Antibiotics can alter host immunity and metabolism, incompletely deplete resident communities, and exert off-target effects that complicate causal interpretation. These models are informative, but they should not be regarded as equivalent substitutes for gnotobiotic systems.

Humanized mouse models may improve translational relevance by partially reconstructing human immune components, but they also remain incomplete. Peripheral-blood-based systems are limited by rapid xenogeneic graft-versus-host disease, whereas hematopoietic stem cell (HSC)-based systems provide broader hematopoietic reconstitution but still rely on non-physiological thymic education and do not fully recapitulate model human mucosal immunity [471]. Even so, no current model fully captures the cellular, ecological, and treatment-related complexity of patient tumors. This limitation should be made explicit when extrapolating preclinical findings to human disease.

Complementing these model systems, genetic labeling and in vivo tracking strategies are beginning to provide a more direct view of microbial dynamics and behavior within tumors. For example, bacteria engineered with fluorescent and selectable cassettes, such as *Staphylococcus xylosus* labelled with erythromycin resistance (erm) and green fluorescent protein (GFP), can be followed during tumor progression and metastatic dissemination [25]. Such approaches are particularly useful for determining whether intratumoral bacteria remain localized, disseminate with tumor cells, or seed distant metastatic sites [472]. In parallel, engineered bacteria used as local delivery vehicles or immunomodulators may offer a more direct means of probing the mechanism while simultaneously testing therapeutic potential [473].

Taken together, progress in this field will depend not only on identifying additional taxa, but also on integrating contamination-aware sequencing, orthogonal validation, and appropriately controlled functional models. Only such a framework can distinguish true intratumoral colonization from background signal and move the field from descriptive association towards mechanism.

## Conclusions and perspectives

Research on the ITM has moved beyond the initial question of whether microbes are present within tumors and is now entering a phase in which their biological significance and clinical relevance must be defined more precisely [474, 475]. Current evidence suggests that ITM is better viewed not as a universal hallmark of cancer, but as a context-dependent component of selected tumor ecosystems, with functions shaped by host background, anatomical site, and therapeutic context [26, 475, 476]. Accordingly, future research needs to move beyond descriptive taxonomic cataloguing and towards defining when, where, and under what conditions ITM becomes functionally relevant [424, 475]. A more localized ecological framework also helps explain early technical artefacts, including the misclassification of human sequences as bacterial signals, and provides a clearer basis for establishing more rigorous biological standards in ITM research.

Despite growing interest in its translational potential, the field remains constrained by technical limitations, fundamental biological uncertainties, and challenges in therapeutic implementation [17, 474, 475]. Technically, the low microbial biomass of many tumor samples makes them especially vulnerable to environmental contamination and host nucleic acid interference, placing stringent demands on sequencing depth and computational decontamination [4, 424, 457, 475]. Biologically, several central questions remain unresolved, including whether ITM seeds tumors early and contributes actively to oncogenesis or instead accumulates passively in advanced lesions, how tumor-associated microbes traffic to and colonize specific tissues, and how fungi, viruses, and archaea, together with bacteria, shape the TIME over time [474–476]. These uncertainties are compounded in translational settings, where live microbial agents differ fundamentally from conventional small-molecule drugs in pharmacokinetic behavior and often exhibit nonlinear dose–response relationships, making optimal dosing regimens and treatment schedules difficult to define [17, 424, 474]. Clinical development is further complicated by safety considerations, as uncontrolled bacterial expansion, systemic infection, cytokine release syndrome (CRS), and the need for Good Manufacturing Practice (GMP)-compliant manufacturing all remain major barriers to implementation [17, 477, 478].

Future progress will therefore depend on the development of more precise and personalized intervention strategies [474, 475]. Successful clinical translation is likely to require closer alignment between therapeutic approaches and the microecological features of individual tumors. One promising direction is the use of single-cell and spatially resolved transcriptomic approaches, including INVADEseq, to define at subcellular resolution how ITM reshapes tumor architecture and immune states, thereby improving patient stratification [462, 479]. Other strategies with substantial translational promise include the selective depletion of drug-resistance-associated bacteria, such as Gammaproteobacteria linked to GEM resistance, as well as the use of synthetic biology to engineer smart-sensing bacteria that release therapeutic payloads selectively in response to features of the TME, including hypoxia, acidity, and elevated lactate [131, 474, 477, 478, 480].

Several questions now warrant particular attention. These include whether endogenous ITM can be monitored in real time through non-invasive circulating microbial signals, for example, by liquid biopsy; how the spatiotemporal dynamics of ITM under chemotherapy or immunotherapy influence long-term clinical outcomes; whether direct feedback loops exist between the gut microbiome and the ITM; and how spatial multi-omics can be integrated with AI-based predictive models to support individualized immunotherapies tailored to microbial profiles. Addressing these questions may help shift oncology away from destructive and non-specific treatment paradigms and towards more selective, biologically responsive, and clinically adaptable forms of precision medicine.

## Data Availability

Not applicable.
